# RNF138 regulates skeletal muscle differentiation via the Wnt/β-catenin signaling pathway

**DOI:** 10.7150/thno.110925

**Published:** 2025-03-18

**Authors:** Wenhao Wang, Zhuohua Wang, Rourong Li, Weiyi Huang, Qiao Ling, Xiaoxiao Li, Zan Li, Manqi Cao, Zhihui Zhang, Qingrong Sun, Zhijuan Liang, Hua-an Zhang, Xuan Jiang, Chuwen Lin, Yaoqing Chen, Bo Zhao, Yu Zhao, Ji-An Pan, Xiaoxue Peng

**Affiliations:** 1 The Molecular Cancer Research Center, School of Medicine, Shenzhen Campus of Sun Yat-sen University, No. 66, Gongchang Road, Guangming District, Shenzhen, Guangdong 518107, China.; 2 The Center for Infection and Immunity Study, School of Medicine, Shenzhen Campus of Sun Yat-sen University, No. 66, Gongchang Road, Guangming District, Shenzhen, Guangdong 518107, China.; 3 Shenzhen Key Laboratory for Systems Medicine in Inflammatory Diseases, School of Medicine, Shenzhen Campus of Sun Yat-sen University, No. 66, Gongchang Road, Guangming District, Shenzhen, Guangdong 518107, China.; 4 School of Public Health (Shenzhen), Shenzhen Campus of Sun Yat-sen University, No. 66, Gongchang Road, Guangming District, Shenzhen, Guangdong 518107, China.

**Keywords:** RNF138, skeletal muscle differentiation, Wnt/β-catenin, adenomatous polyposis coli

## Abstract

**Rationale:** Myogenesis is a strictly regulated process driven by signaling pathways activating muscle-specific gene expression. During myogenesis, muscle stem cells exhibit DNA damage response (DDR) features, which are essential for myoblast differentiation and skeletal muscle regeneration. However, the specific roles of DDR-associated proteins in these processes are not yet fully understood.

**Methods:** Gene knockdown and knockout were used in cell and animal models to study RNF138's function in myoblast differentiation and skeletal muscle regeneration. Multi-omics profiling, including transcriptomics and proteomics, was conducted to identify the key proteins regulated by RNF138 in myogenesis. Protein turnover assays were utilized to investigate RNF138's role in APC protein turnover. Immunofluorescence microscopy was performed to confirm the protein colocalization and subcellular localization.

**Results:** RNF138 expression increases during myoblast differentiation and in regenerating myofibers following muscle injury. Knockdown of RNF138 in C2C12 myoblasts impairs myogenic differentiation and fusion. Additionally, *Rnf138*-deficient mice exhibit delayed muscle regeneration following cardiotoxin-induced injury. Multi-omics profiling, including transcriptomics and proteomics, reveals that Wnt/β-catenin signaling, a key driver of myogenic differentiation, is enhanced by RNF138. Mechanistically, RNF138 stabilizes β-catenin and enhances its nuclear localization by facilitating lysosomal degradation of APC, a component of the β-catenin degradation complex responsible for mediating the export of β-catenin from the nucleus to the cytoplasm for further ubiquitin-proteasome degradation.

**Conclusions:** We reveal a noncanonical role for RNF138, an E3 ubiquitin ligase, as a positive regulator of myoblast differentiation and skeletal muscle regeneration via the Wnt/β-catenin pathway. This finding highlights the noncanonical function of RNF138 beyond its known roles in DDR and other cellular processes. Therefore, RNF138 provides a potential link between DDR and myoblast differentiation, offering new insights into the molecular regulation of muscle regeneration.

## Introduction

Skeletal muscle is a complex and heterogeneous tissue composed of multinucleated myofibers, neurons, blood vessels, lymphatics, and connective tissue 1. It exhibits remarkable regenerative capabilities, relying on muscle-resident stem cells, called satellite cells, to maintain homeostasis in response to muscle injury, disease, or aging. Myofiber maturation initiates as satellite cells exit quiescence, re-enter the cell cycle, and give rise to committed myogenic progenitor cells, or myoblasts. After several rounds of proliferation, myoblasts withdraw from the cell cycle, differentiate, and fuse with each other or existing myofibers to form functional, newly generated myofibers 2, 3.

Myogenic regulatory factors (MRFs), including Myf5, MyoD, Myogenin, and MRF4, are members of the basic helix-loop-helix (bHLH) transcription factor family and play crucial roles in regulating myogenic lineage commitment and differentiation 4. In quiescent satellite cells, Myf5 is predominantly expressed, but its levels decrease during myogenic differentiation, while the expression of Myogenin and MRF4 increases 5, 6. During differentiation, MyoD and Myf5 share approximately 30% of their DNA binding motifs, yet Myf5 has minimal impact on gene activation compared to MyoD, which recruits RNA Polymerase II and activates transcription, including that of *Myogenin* 7, 8. Myogenin triggers the terminal differentiation, followed by the expression of MRF4 during the fusion process 9. Additionally, Myomaker, a recently identified muscle-specific factor expressed during myoblast fusion, is crucial for cell-cell fusion 10.

Wnt signaling plays a pivotal role in muscle formation during both embryonic development and postnatal differentiation 11, 12. In adult skeletal muscle, Wnt signaling regulates myogenic differentiation through the canonical β-catenin-dependent pathway 13, 14. Additionally, it supports satellite cell self-renewal and myofiber hypertrophy via non-canonical β-catenin-independent pathways 15, 16. In the canonical Wnt signaling pathway, Wnt ligands bind to Frizzled receptors, leading to the disruption of the β-catenin destruction complex, which includes Axin, adenomatous polyposis coli (APC), serine-threonine kinase glycogen synthase kinase-3β (GSK-3β), and casein kinase 1α (CK1α) 17. This disruption prevents β-catenin degradation, allowing its translocation into the nucleus, where it activates the β-catenin/TCF complex.

RNF138 is a C3HC4-type RING finger domain E3 ligase with five functional domains: a RING domain, three zinc fingers, and a ubiquitin-interaction motif 18, 19. It is involved in multiple biological processes, including DNA repair 20, 21, tumor progression 22, 23, and spermatogenesis 24. In spermatogenesis, RNF138 is highly expressed, and *Rnf138*-deficient mice show delayed spermatogonia differentiation in juvenile males, accompanied by downregulation of cell cycle and proliferation genes in the testes 24. Myogenesis coordinates cell cycle regulation, DNA repair, and muscle-specific gene expression through multiple mechanisms to prevent the formation of undifferentiated or dysfunctional myofibers, indicating a potential role for RNF138 in myogenic differentiation 25, 26.

In this study, we investigated the physiological role of RNF138 in myogenesis and its impact on muscle regeneration. We revealed that RNF138 stabilizes β-catenin by promoting the lysosomal degradation of APC, which prevents β-catenin's export and degradation, thereby enhancing Wnt/β-catenin signaling activation. Although the precise mechanisms by which RNF138 modulates β-catenin localization still await future investigations, our findings establish RNF138 as a critical regulator of myogenic differentiation and muscle regeneration.

## Results

### RNF138 is crucial for myoblast differentiation

**Figure 1:**
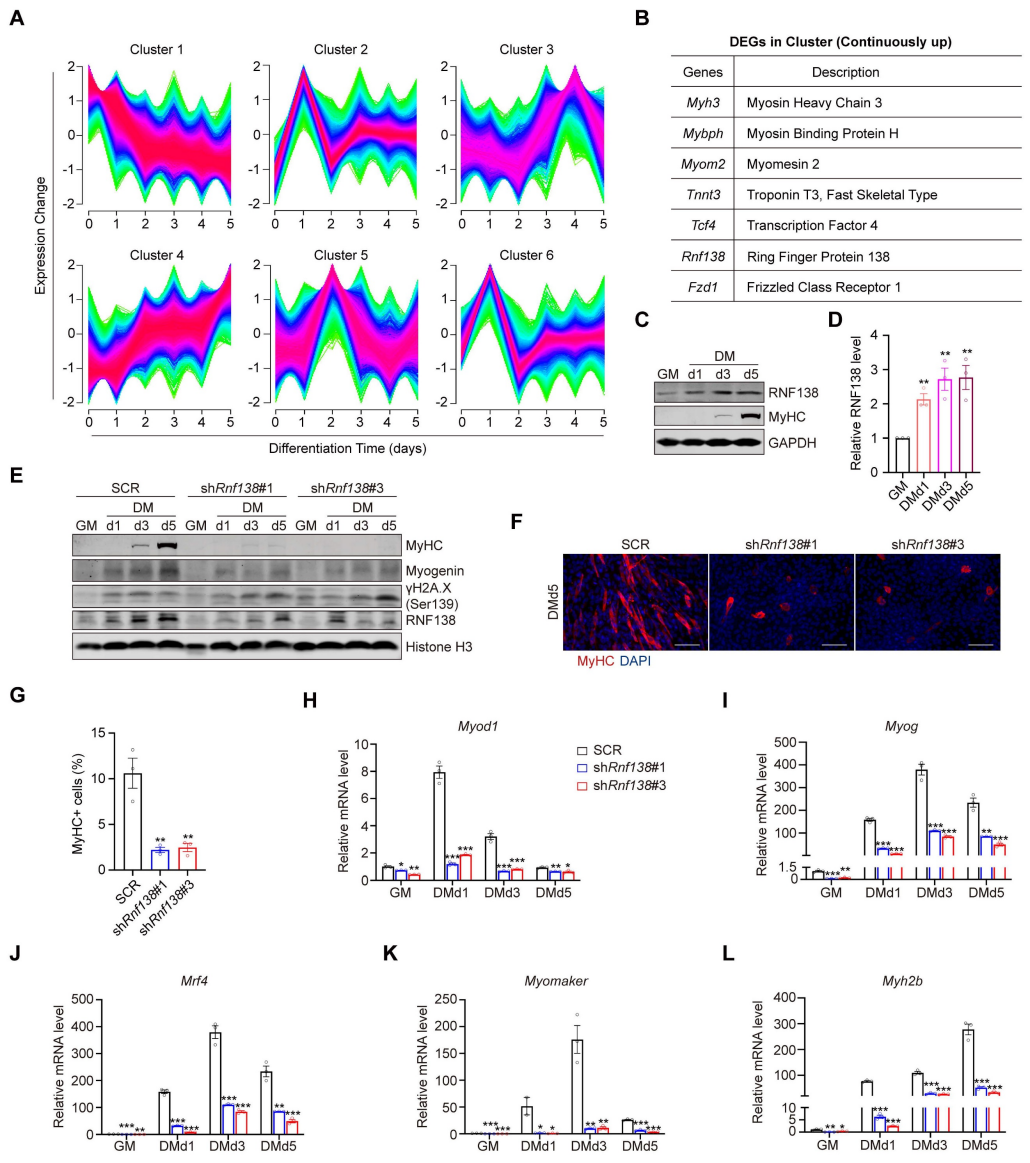
** RNF138 is essential for myoblast differentiation.** (A) Temporal gene expression profiles of six distinct clusters identified by Mfuzz clustering during C2C12 myoblast differentiation. (B) Genes within Cluster 4 that are continuously upregulated during C2C12 myoblast differentiation, including key regulators of myogenic differentiation such as *Myh3* (Myosin Heavy Chain 3), *Mybph* (Myosin Binding Protein H), and *Tnnt3* (Troponin T3, fast skeletal type). (C, D) Western blot analysis showing RNF138 and MyHC protein levels at the indicated time points during C2C12 myoblast differentiation. The quantification of relative RNF138 protein levels during differentiation is shown in Panel (D). RNF138 expression was normalized to GAPDH and presented as fold change relative to GM (growth medium) conditions. Data are shown as mean ± SEM (n = 3 independent experiments). Statistical significance was determined using Student's *t*-test (**p < 0.01). (E) Western blot analysis of MyHC expression in scrambled control (SCR) and *Rnf138*-knockdown (*Rnf138*-KD) C2C12 myoblasts during differentiation. (F) Representative immunofluorescence staining images of MyHC (red) and nuclei (DAPI, blue) in C2C12 myoblasts after 5 days of differentiation. Scale bar: 100 µm. (G) Quantification of the fusion index, defined as the percentage of nuclei within MyHC-positive multinucleated myotubes. Data are presented as mean ± SEM (n = 3 fields per group). Statistical significance was determined using Student's *t*-test, comparing each *Rnf138*-KD group to the SCR group (**p < 0.01). (H-L) Bar plots of mRNA expression levels of myogenic genes in *Rnf138*-KD and SCR C2C12 myoblasts at the indicated time points during differentiation. (H) *Myod1*, (I) *Myog*, (J) *Mrf4*, (K) *Myomaker*, and (L) *Myh2b*. Data are normalized to the mRNA levels in the SCR-GM group and presented as mean ± SEM from three independent experiments. Statistical significance was determined by Student's *t*-test, comparing each *Rnf138*-KD group to the SCR group at each time point (*p < 0.05, **p < 0.01, ***p < 0.001).

To investigate the potential role of RNF138 in myogenesis, we analyzed its expression profile during the differentiation of C2C12 myoblast. A time-course analysis of the Gene Expression Omnibus (GEO) dataset GSE11415 identified six clusters with distinct temporal gene expression patterns during the differentiation process. The mRNA expression of *Rnf138* was found to be enriched in cluster 4, where genes exhibit continuous upregulation throughout differentiation (Figure 1A-B). To validate this expression pattern, we induced differentiation in C2C12 myoblasts and observed a steady increase in RNF138 expression alongside myosin heavy chain (MyHC), suggesting a potential role of RNF138 in myogenic differentiation (Figure 1C-D).

To confirm the role of RNF138 in myoblast differentiation, we generated stable *Rnf138* knockdown *(Rnf138*-KD) C2C12 myoblasts using lentivirus-mediated shRNA interference (Figure S1). As a result, the downregulation of RNF138 led to a marked reduction in the expression of myogenic markers, including MyHC and Myogenin, at all examined time points during differentiation, compared to the scrambled control (SCR) group (Figure 1E). Notably, downregulation of RNF138 results in incomplete DNA damage repair, as evidenced by elevated levels of phosphorylated H2AX (γH2AX) (Figure 1E). This highlights that RNF138's role in myoblast differentiation is closely linked to its function in DNA repair, which is essential for maintaining genomic stability during this process 21, 25. Immunofluorescent (IF) staining of MyHC revealed that *Rnf138*-KD caused a nearly complete loss of cell-cell fusion and MyHC-positive myofiber formation (Figure 1F-G). Consistent with these findings, mRNA levels of key myogenic regulatory genes encoding MyoD, Myogenin, MRF4, Myomaker, and MyHC, were markedly reduced in *Rnf138*-KD C2C12 myoblasts (Figure 1H-L). These genes are essential for myoblast commitment and fusion, underscoring the importance of RNF138 in this process.

### RNF138 modulates the expression of myogenic genes

**Figure 2:**
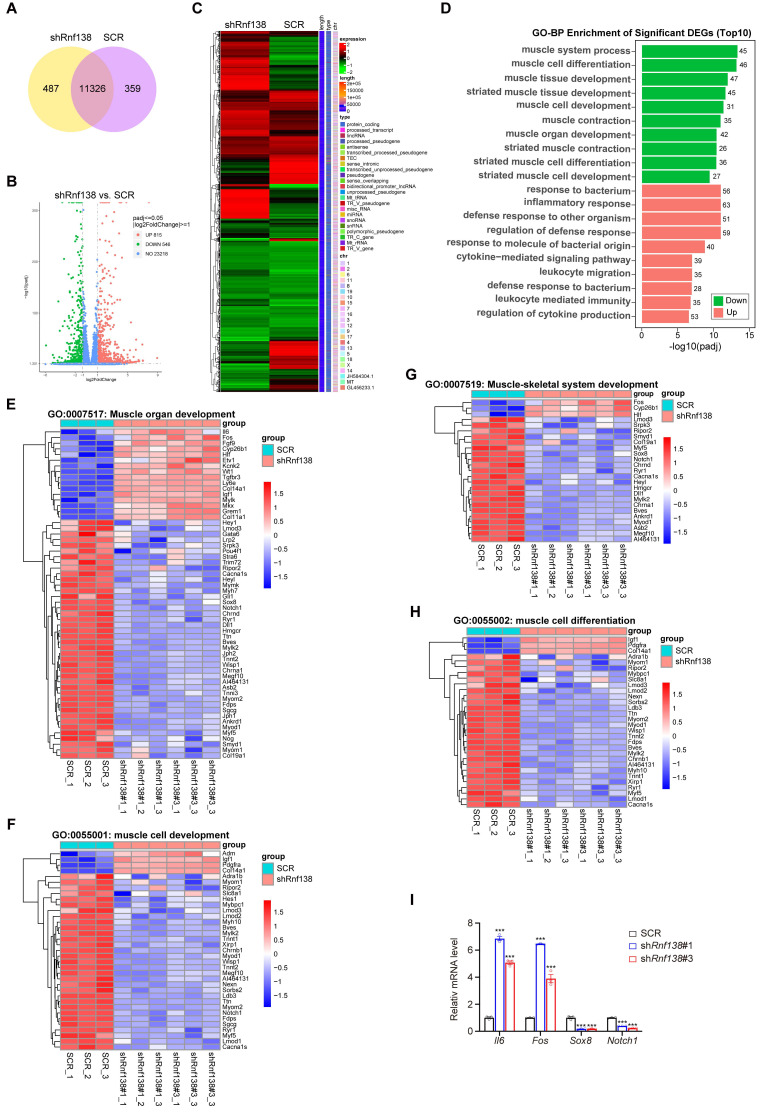
** Clustering analysis of differentially expressed genes (DEGs) following *Rnf138*-knockdown (*Rnf138*-KD) in differentiating C2C12 myoblasts.** (A) Venn diagram illustrating the overlap and unique gene expression profiles between *Rnf138*-KD and scrambled control (SCR) C2C12 myoblasts. Numbers indicate unique and shared genes between the two groups. (B) Volcano plot showing the distribution of DEGs between the *Rnf138*-KD and SCR groups. DEGs with statistical significance (adjusted *p*-value < 0.05) and log2(Fold Change) > 1 are highlighted in green (downregulated) and red (upregulated). (C) Heatmap displaying hierarchical clustering of DEGs between *Rnf138*-KD and SCR myoblasts. Red represents upregulated genes, and green represents downregulated genes. (D) Gene Ontology-Biological Process (GO-BP) enrichment analysis of DEGs, highlighting the top 10 enriched GO terms. Numbers on the bars indicate the DEG counts within each category. (E-H) Heatmaps showing the expression levels of genes in muscle-related GO categories. (E) Muscle organ development (GO:0007517), (F) Muscle cell development (GO:0055001), (G) Muscle-skeletal system development (GO:0007519), and (H) Muscle cell differentiation (GO:0055002). (I) Quantitative RT-PCR analysis of *Il6, Fos, Sox8, and Notch1* mRNA expression levels in *Rnf138*-KD and SCR myoblasts at the indicated time points. Data are presented as mean ± SEM from three independent experiments. Statistical significance was determined by Student's *t*-test (***p < 0.001).

In our subsequent investigation, we examined the impact of RNF138 knockdown on the transcriptional regulation of myogenic genes in C2C12 myoblasts. A Venn diagram was employed to illustrate the overlap and unique gene expression profiles between the RNF138 knockdown and control myoblasts, with 487 genes unique to the RNF138 knockdown group, 359 unique to the control group, and 11,326 shared by both groups (Figure 2A). Specifically, a total of 1,361 differentially expressed genes (DEGs) were identified with statistical significance between the RNF138 knockdown and control groups (Figure 2B-C). Among these DEGs, 546 genes were downregulated, while 815 genes were upregulated.

Gene ontology (GO) analysis of the DEGs highlighted significant involvement of downregulated genes in muscle-related biological processes, such as muscle system process, muscle cell differentiation, and muscle cell development (Figure 2D). This suggests that RNF138 supports myogenic differentiation at the transcriptional level. In contrast, upregulated genes were enriched in immune-related processes, including inflammatory response and response to bacterial origin. This shift implies that RNF138 knockdown may alter the balance between immune activation and myogenic differentiation, potentially impairing muscle cell development (Figure 2D) 27, 28.

Detailed heatmaps of key GO categories further illustrate the differential expression within these functional groups (Figure 2E-H). For groups such as “muscle organ development” (Figure 2E), “muscle cell development” (Figure 2F), “muscle-skeletal system development” (Figure 2G), and “muscle cell differentiation” (Figure 2H), key genes involved in myogenesis were significantly downregulated in RNF138 knockdown myoblasts compared to controls, aligning with previous findings (Figure 1H-L). Furthermore, immune-related genes exhibited notable upregulation in the RNF138 knockdown group. For instance, *Il6* (Interleukin-6) 29 and *Fos* 30 are known to play critical roles in immune responses and inflammation. *Il6* functions as a pro-inflammatory cytokine, whereas *Fos*, which encodes the transcription factor c-Fos, is implicated in inflammation and stress responses. Additional upregulated immune-related genes include *Tgfbr3* (Transforming Growth Factor Beta Receptor 3) 31, involved in TGF-β signaling and immune modulation, and *Col14a1* 32 and *Col11a1* 33, which, while primarily structural components, may contribute to tissue remodeling and inflammatory responses following RNF138 knockdown. In line with these results, quantitative PCR analysis confirmed the upregulation of *Il6* and *Fos* mRNA levels in RNF138 knockdown myoblasts compared to SCR cells (Figure 2I). Conversely, myogenesis-related genes such as *Sox8* 34 and *Notch1* 35 were significantly downregulated. Sox8, a member of the SOX family of transcription factors, plays a pivotal role in muscle progenitor cell differentiation and the regulation of muscle cell lineage commitment. Its reduced expression in RNF138 knockdown cells suggests a disruption in the early differentiation processes of myoblasts. Similarly, Notch1, a critical component of the Notch signaling pathway, is well-known for its involvement in maintaining the balance between myoblast proliferation and differentiation 36.

Consistent with these observations in the differentiation phase, RNF138 knockdown in proliferative C2C12 myoblasts also resulted in downregulation of muscle-related genes and upregulation of immune-related genes, as illustrated in Figure S2, further underscoring RNF138's regulatory role across both phases of muscle development. These contrasting patterns underscore RNF138's dual role in promoting myogenic differentiation while suppressing excessive inflammatory responses. Collectively, these findings suggest that RNF138 is essential for maintaining a balanced gene expression network that supports myoblast differentiation while preventing detrimental immune activation during muscle development.

### RNF138 is essential for muscle regeneration

**Figure 3:**
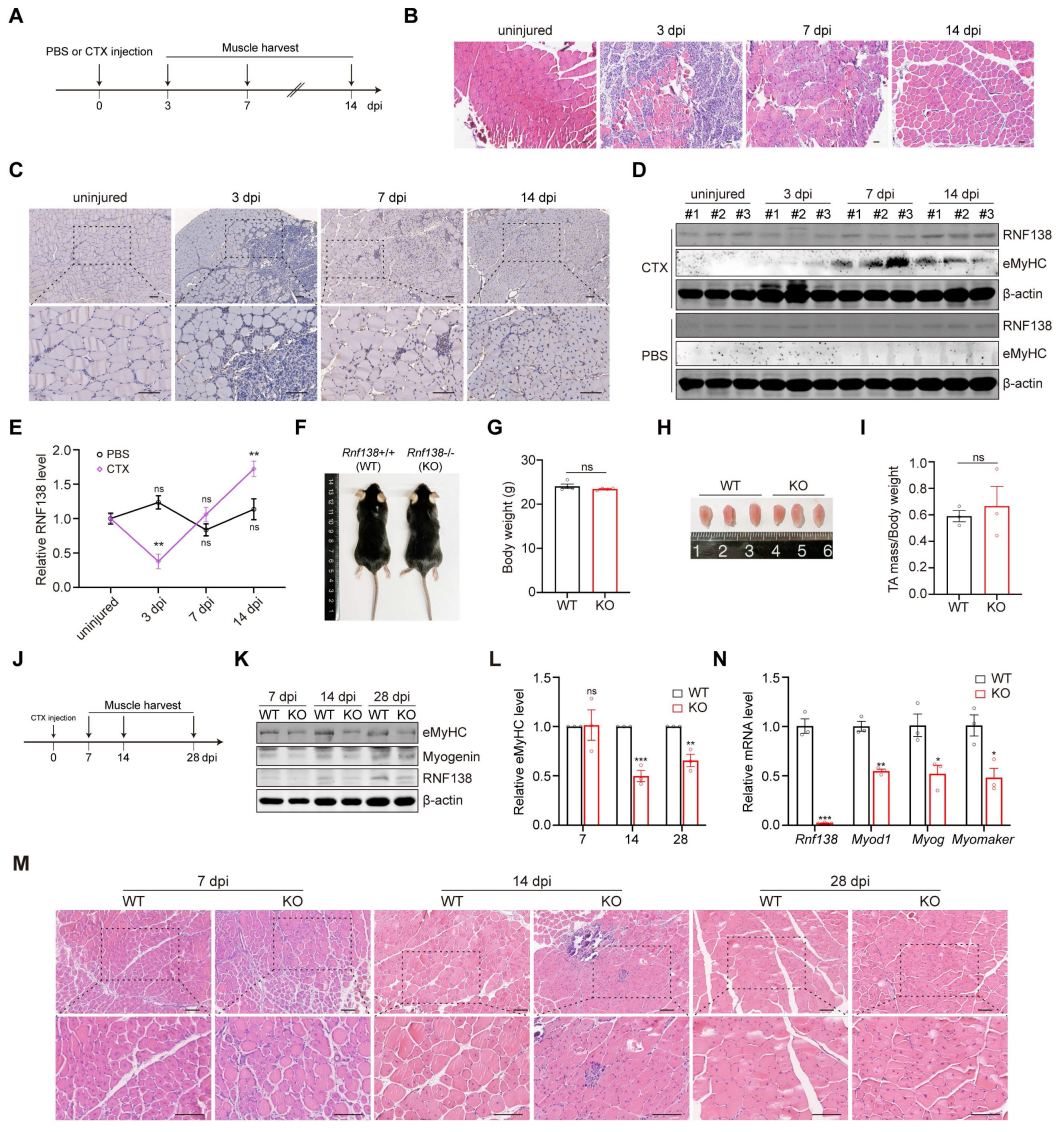
** RNF138 is required for muscle regeneration.** (A) Schematic representation of the experimental design for muscle injury and regeneration analysis. Tibialis anterior (TA) muscles were injected with PBS or cardiotoxin (CTX) at day 0, followed by tissue harvest at 3-, 7-, and 14-days post-injury (dpi). Each group consisted of three mice (n = 3). (B) Hematoxylin and eosin (H&E) staining of TA muscle sections at the indicated time points. Scale bar: 100 μm. (C) Immunohistochemistry (IHC) staining of RNF138 in TA muscle sections at the indicated time points. Lower panels show magnified views of the boxed regions in the upper panels. Scale bar: 100 μm. (D, E) Western blot analysis of RNF138 protein levels in injured (CTX-injected) and control (PBS-injected) TA muscles at the indicated time points, with embryonic myosin heavy chain (eMyHC) used as a marker of muscle regeneration. β-actin was used as a loading control. The quantification of the Western blot results is shown in Panel (E), where relative RNF138 expression levels are plotted for each time point, with comparisons made to the uninjured group. Data represent mean ± SEM (n = 3), and statistical significance was determined by Student's *t*-test (**p < 0.01, ns: not significant). (F) Representative images of WT and *Rnf138*-knockout (*Rnf138*-KO) mice at 10 weeks old under basal conditions. (G) Body weight measurements of WT and *Rnf138*-KO mice under basal (uninjured) conditions (n = 3 per group). Data represent the mean ± SEM. Statistical significance was determined by Student's *t*-test (ns: not significant). (H) Representative images of TA muscles from WT and *Rnf138*-KO mice at 10 weeks old under basal conditions. (I) TA muscle mass normalized to body weight in WT and *Rnf138*-KO mice at 10 weeks old under basal conditions. Data represent mean ± SEM (n = 3), and statistical significance was determined by Student's *t*-test (ns: not significant). (J) Schematic representation of the experimental procedure to compare muscle regeneration between WT and *Rnf138*-KO mice following CTX injection. TA muscles were harvested at 7, 14 and 28 dpi for histological and molecular analyses. (K, L) Western blot analysis of eMyHC, myogenin, β-catenin, and RNF138 protein levels in TA muscles from WT and *Rnf138*-KO mice at 7, 14 and 28 dpi. β-actin was used as a loading control. The quantification of eMyHC levels relative to β-actin is shown in Panel (L), with data presented as mean ± SEM (n = 3). Statistical significance was determined by Student's *t*-test (**p < 0.01, ***p < 0.001, ns: not significant). (M) H&E staining of regenerating TA muscle sections at 7, 14 and 28 dpi. Lower panels provide magnified views of the boxed regions in the upper panels. Scale bar: 100 μm. (N) Quantitative RT-PCR analysis of *Rnf138*, *Myod1*, *Myogenin* (*Myog*), and *Myomaker* mRNA levels in TA muscle tissues from WT and *Rnf138*-KO mice at 14 dpi. Data represent the mean ± SEM from three biological replicates (n = 3). Statistical significance was determined by Student's *t*-test, comparing the KO group to the WT group (*p < 0.05, **p < 0.01, ***p < 0.001).

To investigate the role of RNF138 in muscle injury repair, we induced acute muscle damage in the tibialis anterior (TA) muscle of mice through cardiotoxin (CTX) injection and monitored the expression of RNF138 during the repair process. The experimental design is illustrated in Figure 3A. Hematoxylin and eosin (H&E) staining confirmed substantial muscle fiber damage occurred following CTX injection, with signs of tissue repair visible at subsequent time points (Figure 3B). Immunohistochemistry (IHC) staining revealed sustained expression of RNF138 specifically in regenerating muscle fibers within the damaged areas. In contrast, undamaged fibers in adjacent regions displayed minimal or no RNF138 expression, serving as a baseline for comparison and further emphasizing the injury-induced upregulation of RNF138 (Figure 3C). Western blot analysis, using embryonic myosin heavy chain (eMyHC) as a regeneration marker, further validated the increase in RNF138 levels following injury (Figure 3D-E). These findings establish RNF138 as a regeneration-associated protein upregulated in regenerating myofibers.

To confirm the physiological role of RNF138 in muscle regeneration, mice with *Rnf138*-knockout (KO) were generated using CRISPR/Cas9 technology targeting exon3 of *Rnf138* gene (Figure 3K and 3N). Under basal (uninjured) conditions, *Rnf138*-KO mice exhibited body weights comparable to wild-type (WT) mice, with no significant differences observed (Figure 3F-G). Similarly, gross examination and measurements of TA muscle mass normalized to body weight revealed no notable disparities between WT and KO mice (Figure 3H-I).

To assess the regenerative capacity of skeletal muscles, both WT and *Rnf138*-KO mice underwent identical experimental procedures, with CTX injection performed in the TA muscles. Muscle samples were subsequently collected at 7, 14 and 28 days post-injection (dpi) as illustrated in Figure 3J. As expected, a delayed skeletal muscle regeneration process was observed in *Rnf138*-KO mice compared to WT mice. Western blot analysis revealed significantly reduced protein levels of eMyHC and Myogenin in *Rnf138*-KO mice at all observed time points, indicating a delayed regeneration process and impaired expression of regeneration-associated proteins (Figure 3K-L). H&E staining was performed to examine the histological features of regenerating muscle in WT and *Rnf138*-KO mice (Figure 3M). At 7 dpi, no significant differences were observed between WT and *Rnf138*-KO muscles, as both were still in the early stages of repair, characterized by minimal signs of regeneration and ongoing inflammation. At 14 dpi, WT muscle exhibited robust signs of regeneration, characterized by the presence of numerous newly formed myofibers containing centrally located nuclei, indicative of active repair and myofiber maturation. In contrast, *Rnf138*-KO muscle showed delayed regeneration, characterized by fewer regenerating myofibers, smaller fiber size, and heightened inflammatory cell infiltration. By 28 dpi, WT muscle had progressed significantly, with larger myofibers and many nuclei migrating to the periphery, reflecting advanced maturation. However, *Rnf138*-KO mice displayed persistent delays, as evidenced by smaller myofibers with a higher proportion of centrally located nuclei, suggesting impaired myofiber maturation and incomplete repair. In parallel, qRT-PCR analysis demonstrated markedly lower transcript levels of key myogenic markers, including *Myod1*, *Myog*, and *Myomaker*, in *Rnf138*-KO mice at 14 dpi relative to WT controls (Figure 3N).

Collectively, these findings underscore the essential role of RNF138 in facilitating efficient skeletal muscle regeneration and promoting timely myofiber maturation following injury.

### RNF138 regulates myogenic differentiation via targeting β-catenin

Previous studies have demonstrated that RNF138 is involved in the DNA damage response by mediating the ubiquitylation of CtBP-interacting protein (CtIP) and RAD51D, with a particular affinity for resected double-stranded DNA (dsDNA) containing single-stranded DNA (ssDNA) overhangs, but not blunt-ended dsDNA 20, 21, 37. This functional association with DNA processing and repair highlights its potential involvement in nuclear regulatory mechanisms. To clarify whether RNF138 directly regulates myogenic gene expression, we performed chromatin immunoprecipitation (ChIP) assays. However, RNF138 did not exhibit binding to classical target genes such as *RPL30*, *GAPDH*, *RPL13A*, and *MYC* (data not shown). This aligns with our understanding that RNF138 does not possess the capacity to bind DNA sequences directly 21. These findings collectively indicate that RNF138 likely exerts its effects through downstream effectors, potentially by modulating signaling cascades, protein localization, or post-translational modifications.

To identify potential downstream targets of RNF138, we analyzed its interactome using co-immunoprecipitation followed by mass spectrometry (Co-IP/MS). Enrichment analysis revealed that RNF138-interacting proteins are involved in key cellular processes, including cell growth, nucleic acid metabolism, and protein metabolism (Figure S3A), suggesting a broad regulatory impact. Functionally, RNF138-interacting proteins were enriched in nucleic acid binding and ubiquitin-related activities (Figure S3B), highlighting its involvement in RNA and protein regulation. Cellular component analysis shows RNF138's presence across multiple cellular compartments, including the cytoplasm, nucleus, and nucleolus (Figure S3C), suggesting its influence is widespread within the cell.

**Figure 4:**
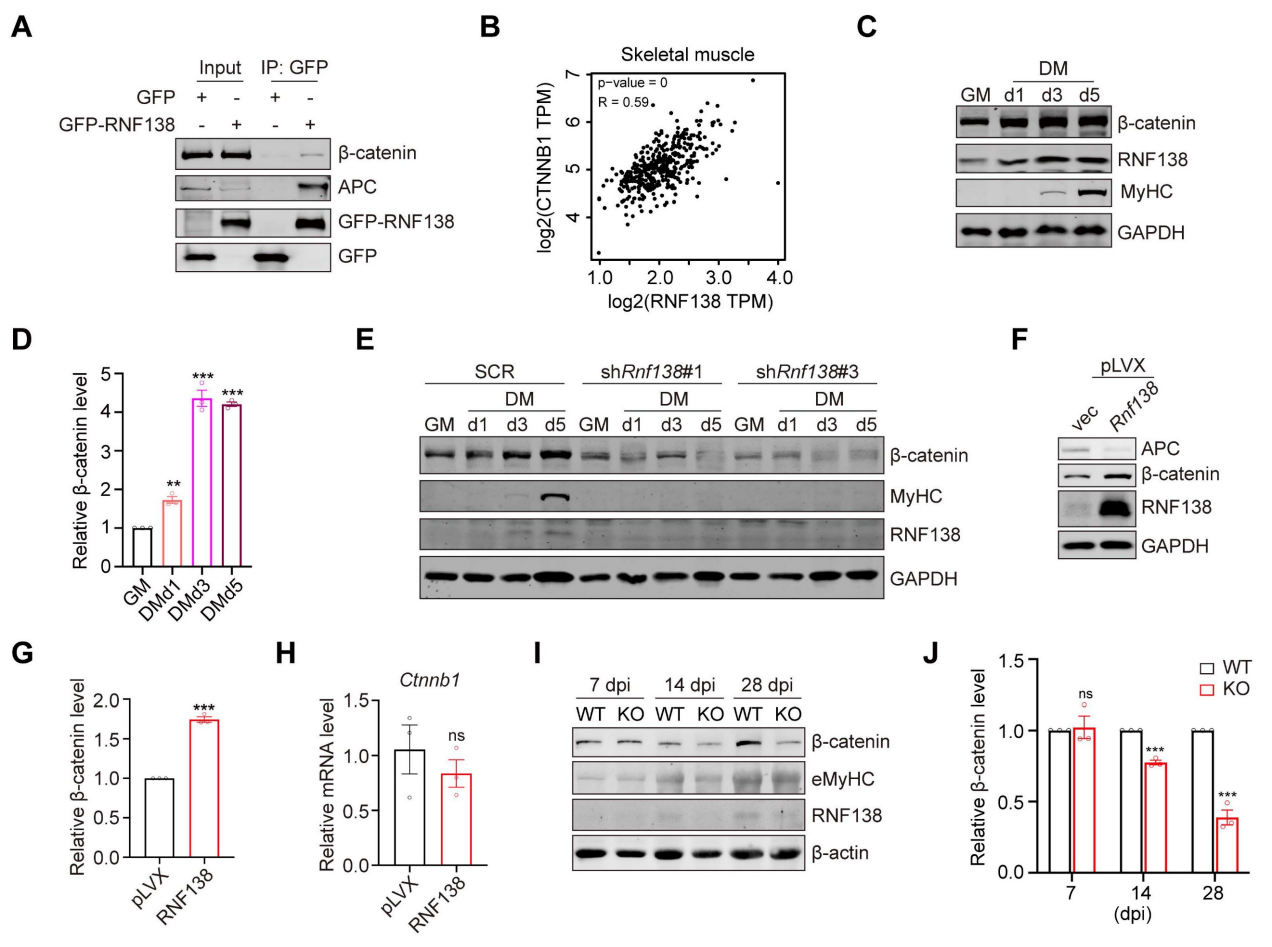
** β-catenin is a downstream target of RNF138.** (A) Co-immunoprecipitation (Co-IP) assays were performed on protein extracts from HEK-293T cells transfected with GFP-RNF138 or GFP expression vector to detect interactions between RNF138, β-catenin, and APC. GFP-nanobeads were used for immunoprecipitation (IP), and the IP samples were examined by immunoblotting (IB) with antibodies against β-catenin, APC, and GFP. (B) Correlation analysis between RNF138 (*RNF138*) and β-catenin (*CTNNB1*) mRNA expression levels in skeletal muscle tissues using the GEPIA database (http://gepia.cancer-pku.cn/). (C, D) Western blot analysis of β-catenin, RNF138, and MyHC expression in C2C12 myoblasts during proliferation (GM) and differentiation (DM) at day 1, 3, and 5. The quantification of relative β-catenin protein levels during myoblast differentiation is shown in Panel (D). β-catenin expression was normalized to GAPDH and presented as fold change relative to GM (growth medium) conditions. Data are shown as mean ± SEM (n = 3 independent experiments). Statistical significance was determined using Student's *t*-test (**p < 0.01, ***p < 0.001). (E) Western blot analysis of β-catenin, RNF138, and MyHC expression in scrambled control (SCR) and *Rnf138*-knockdown (*Rnf138*-KD) C2C12 cells during proliferation (GM) and differentiation (DM) at days 1, 3, and 5. (F, G) Western blot analysis of APC and β-catenin expression in C2C12 myoblasts stably overexpressing RNF138 or vector control. The quantification of relative β-catenin protein levels is shown in Panel (G), comparing RNF138-overexpressing cells to the vector control. Data are presented as mean ± SEM from three independent experiments. Statistical significance was determined by Student's *t*-test (***p < 0.001). (H) Relative mRNA expression levels of *Ctnnb1* in C2C12 myoblasts with RNF138 overexpression compared to vector control. Data are presented as mean ± SEM from three independent experiments. Statistical significance was determined by Student's *t*-test (ns: not significant). (I, J) Western blot analysis of β-catenin, eMyHC, RNF138 protein levels in TA muscles from WT and *Rnf138*-KO mice at 7, 14 and 28 dpi. β-actin was used as a loading control. The quantification of β-catenin levels relative to β-actin is shown in Panel (J), with data presented as mean ± SEM (n = 3). Statistical significance was determined by Student's *t*-test (***p < 0.001, ns: not significant).

Among RNF138's interacting partners, we identified β-catenin, a key transcriptional co-activator in the Wnt signaling pathway that plays critical roles in skeletal muscle formation and tissue homeostasis maintenance 13. We confirmed the interaction between RNF138 and β-catenin (Figure 4A) and further explored their correlation in myoblast differentiation. Analysis within the Gene Expression Profiling Interactive Analysis (GEPIA) online tool 38 showed a positive transcriptional correlation between RNF138 and the β-catenin encoding gene CTNNB1 in skeletal muscle tissues (Figure 4B). Similar to RNF138, β-catenin expression progressively increased during differentiation, peaking on day 3 (Figure 4C-D), indicating that RNF138 may influence β-catenin expression and/or stability, thereby impacting myogenic gene expression.

The Wnt/β-catenin signaling pathway is well-established as essential for skeletal muscle regeneration. We confirmed this by demonstrating that *Ctnnb1* knockdown (*Ctnnb1*-KD) impairs C2C12 myoblast differentiation (Figure S4). We generated C2C12 cell lines with *Ctnnb1* knockdown and validated the reduced β-catenin levels via western blot and qRT-PCR (Figure S4A-B). As expected, downregulation of β-catenin resulted in a marked reduction in MyHC expression (Figure S4C) and myogenic differentiation (Figure S4D-E) after 5 days of differentiation, as well as decreased expression of *Myod1*, *Myogenin*, *Myomaker*, and *Myh2b* (Figure S4F). Consistent with these findings, pharmacological inhibition of Wnt/β-catenin signaling by XAV939 suppressed C2C12 myoblast differentiation (Figure S4G). Notably, downregulation of β-catenin did not significantly alter RNF138 levels, suggesting that RNF138 functions upstream of β-catenin (Figure S4C).

Thus, we examined β-catenin expression levels in both control and *Rnf138*-KD C2C12 myoblasts. In line with our hypothesis, RNF138 downregulation resulted in decreased β-catenin expression during both proliferation and differentiation (Figure 4E), whereas RNF138 overexpression led to an increase in β-catenin levels (Figure 4F-G). However, analysis of *Ctnnb1* mRNA expression revealed no significant changes, indicating that RNF138 regulates β-catenin protein stability rather than its transcription (Figure 4H).

To validate these findings in a different cellular context, we assessed β-catenin expression in A549 cells, which have a relatively higher RNF138 expression compared with myoblasts. Downregulation of RNF138 in A549 cells led to a reduction in β-catenin levels (Figure S5A), while transient RNF138 overexpression increased β-catenin expression in a time-dependent manner (Figure S5B). However, this increase did not result in a significant enhancement of β-catenin's transcriptional activity (Figure S5C), a finding that is consistent with the results obtained from stable overexpression (Figure 4F), further suggesting that RNF138 primarily functions to stabilize β-catenin, rather than directly modulating its transcriptional activity. Given RNF138's established role in DNA damage repair, we further investigated the RNF138-β-catenin relationship under DNA damage conditions. Treatment with arsenate (As) in HEK293T, U2OS, and C2C12 cells induced DNA damage, as indicated by histone H2A.X phosphorylation (γ-H2A.X), and was accompanied by increased RNF138 and β-catenin levels (Figure S5D). Similar results were observed with etoposide (Eto) treatment, reinforcing the association between RNF138 and β-catenin (Figure S5E).

To further investigate the role of RNF138 in regulating β-catenin during muscle regeneration, we extended our analysis to *in vivo* muscle tissue from both WT and *Rnf138*-KO mice, following the procedure outlined in Figure 3J. Western blot analysis revealed significantly reduced levels of β-catenin in *Rnf138*-KO muscles, particularly at 14 and 28 dpi, corresponding to the later stages of the regeneration process (Figure 4I-J). These findings are consistent with our cell-based experiments, where downregulation of RNF138 resulted in decreased β-catenin expression in C2C12 myoblasts during both proliferation and differentiation (Figure 4E), further supporting the notion that RNF138 is crucial for maintaining β-catenin stability in muscle regeneration.

These findings expand our understanding of RNF138's role in myogenesis by revealing its ability to regulate β-catenin stability. This regulatory mechanism may serve as a potential target for therapeutic strategies in muscle regeneration disorders.

### RNF138 facilitates β-catenin nuclear localization

**Figure 5:**
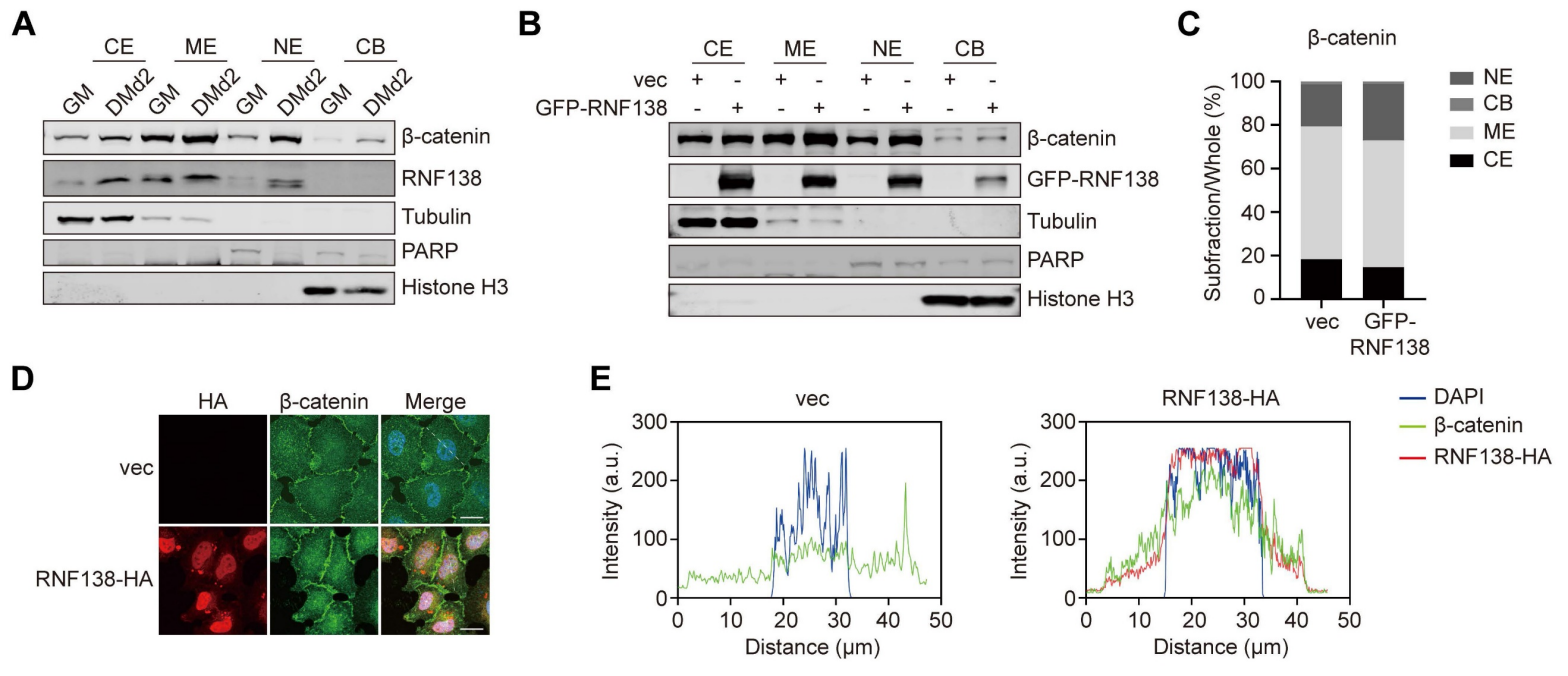
** RNF138 facilitates β-catenin nuclear localization.** (A) Western blot analysis of β-catenin and RNF138 in subcellular fractions (cytoplasmic extract [CE], membrane extract [ME], nuclear extract [NE], and chromatin-bound fraction [CB]) of C2C12 myoblasts during proliferation (GM) and differentiation day 2 (DMd2). Tubulin, PARP, and Histone H3 were used as fraction-specific markers. Tubulin, PARP, and Histone H3 were used as fraction-specific markers, where Tubulin corresponds to the cytosolic extract, PARP to the soluble nuclear extract, and Histone H3 to the chromatin-bound fraction. (B) Western blot analysis of β-catenin and GFP-RNF138 in subcellular fractions of HEK-293T cells transfected with GFP-RNF138 or vector control (vec). Fraction-specific markers are described as Panel (A). (C) Quantitative analysis of β-catenin distribution in different subcellular fractions (CE, ME, NE, CB) in (B). Bars represent the percentage of β-catenin in each fraction relative to the total β-catenin across all fractions. (D) Immunofluorescence (IF) staining of β-catenin and HA-RNF138 in HeLa cells transfected with vector control (vec) or HA-RNF138. Scale bar: 20 μm. (E) Line-scan intensity profiles of DAPI (blue), β-catenin (green), and HA-RNF138 (red) along the nuclear axis, corresponding to images in Panel (D). The white dashed line in Panel (D) indicates the position of the line-scan.

A key characteristic of Wnt/β-catenin signaling activation is the nuclear translocation of β-catenin, which leads to the activation of specific target genes. We assessed β-catenin's subcellular localization during myoblast differentiation and found that cells in the differentiation phase exhibited a notable increase in β-catenin nuclear localization compared to those in the proliferative phase (Figure 5A). This was accompanied by increased nuclear RNF138 levels, although RNF138 was absent from the chromatin-bound fraction, supporting the idea that RNF138 likely regulates myogenesis through downstream effectors, rather than directly binding to myogenic gene promoters.

To further evaluate RNF138's role in β-catenin nuclear translocation, we overexpressed RNF138 in HEK-293T cells, resulting in elevated nuclear β-catenin levels (Figure 5B). Quantitative analysis revealed a proportional increase in the nuclear β-catenin-to-total β-catenin ratio (Figure 5C), and IF staining confirmed RNF138's role in facilitating β-catenin nuclear localization (Figure 5D-E). These results confirm that RNF138 promotes the nuclear translocation of β-catenin.

Additionally, GO analysis of canonical Wnt pathway-related genes differentially expressed upon RNF138 knockdown further supports RNF138's regulatory influence on the Wnt/β-catenin pathway. In the differentiation phase, genes associated with non-canonical Wnt signaling, such as *Prickle1* and *Dact1*, were upregulated, while canonical pathway components, including *Wnt4* and *Fzd9*, were downregulated, suggesting a possible shift towards pathways less conducive to myogenic differentiation (Figure S6A). During the proliferative phase, similar shifts were observed, with the upregulation of Wnt pathway antagonists like *Sfrp1* and *Prickle1* and downregulation of canonical pathway genes, indicating that RNF138's modulation of Wnt signaling may be essential for both myoblast proliferation and differentiation (Figure S6B).

Together, these findings highlight RNF138 as an upstream regulator within the Wnt/β-catenin pathway.

### RNF138 stabilizes β-catenin through promoting lysosomal degradation of APC

**Figure 6:**
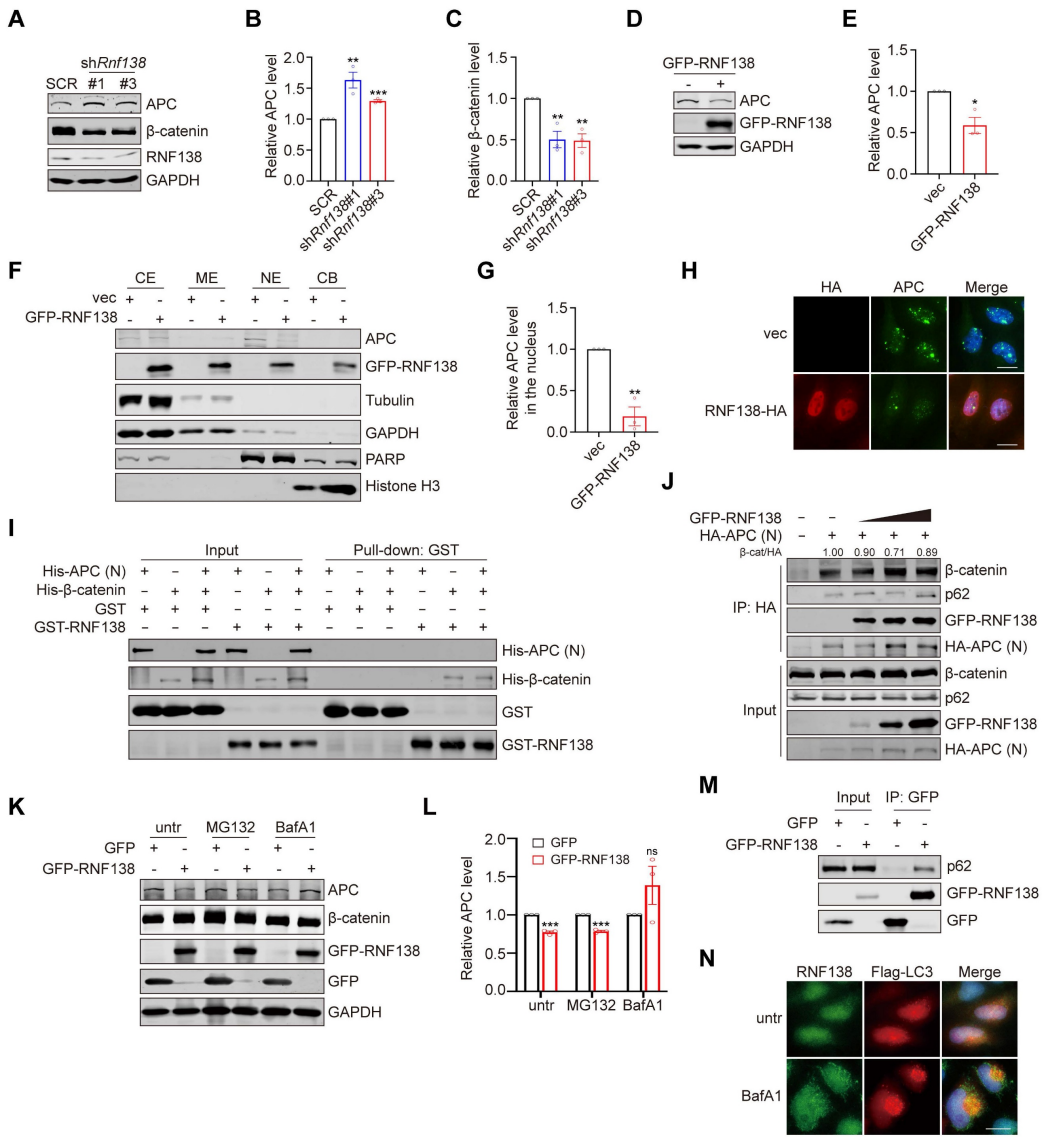
** RNF138 regulating β-catenin through targeting APC.** (A-C) Western blot analysis of APC, β-catenin, and RNF138 expression levels in *Rnf138*-knockdown (*Rnf138*-KD) and scrambled control (SCR) C2C12 myoblasts. The quantification of APC (B) and β-catenin (C) protein levels relative to GAPDH is shown, with data presented as fold change relative to the SCR group. Data are shown as mean ± SEM (n = 3 independent experiments). Statistical significance was determined using Student's *t*-test (**p < 0.01, ***p < 0.001). (D, E) Western blot analysis of APC and GFP-RNF138 expression in HEK-293T cells transfected with either GFP-RNF138 or a control vector (vec). GAPDH was used as a loading control. The quantification of APC levels relative to GAPDH is shown in panel (E), with data presented as mean ± SEM (n = 3). Statistical significance was determined by Student's *t*-test (*p < 0.05). (F, G) Western blot analysis of APC and GFP-RNF138 in subcellular fractions (cytoplasmic extract [CE], membrane extract [ME], nuclear extract [NE], and chromatin-bound fraction [CB]) of HEK-293T cells transfected with or without GFP-RNF138. Fraction-specific markers include Tubulin and GAPDH for the cytosolic extract, PARP for the soluble nuclear extract, and Histone H3 for the chromatin-bound fraction. The quantification of APC levels in the nuclear extract relative to PARP is shown in Panel (G), with data presented as mean ± SEM (n = 3). Statistical significance was determined by Student's *t*-test (**p < 0.01). (H) Immunofluorescence (IF) staining of APC and HA-RNF138 in HeLa cells transfected with vector control (vec) or HA-RNF138. Scale bar: 20 μm. (I) *In vitro* GST-pull-down assay performed with recombinant His-APC (N-terminal), His-β-catenin, and GST-RNF138 proteins. Protein mixtures were incubated with GST beads and precipitated fractions were immunoblotted with the indicated antibodies. (J) Co-immunoprecipitation (co-IP) analysis of β-catenin, GFP-RNF138, and HA-APC (N-terminal) interactions in HEK293T cells. Increasing amounts of GFP-RNF138 were transfected. HA-tagged APC was immunoprecipitated, and β-catenin levels were quantified relative to HA-APC (ratios are indicated). (K, L) Western blot analysis of APC and β-catenin expression in HEK-293T cells transfected with GFP-RNF138 or GFP expression vector following treatment with proteasome inhibitor MG132 or lysosome inhibitor bafilomycin A1 (BafA1). Panel (L) shows the quantification of relative APC levels, normalized to GAPDH, comparing GFP-RNF138 and GFP expression groups under each treatment condition. Data are presented as mean ± SEM (n = 3). Statistical significance was determined using Student's *t*-test (***p < 0.001, ns: not significant). (M) Co-IP analysis was performed on protein extracts from HEK-293T cells transfected with GFP-RNF138 or GFP expression vector to detect interaction between RNF138 and p62. GFP-nanobeads were used for IP, and the IP samples were examined by immunoblotting (IB) with antibodies against p62 and GFP. (N) IF staining of RNF138 and Flag-LC3 in HeLa cells transfected with HA-RNF138. Cells were treated with Bafilomycin A1 (BafA1) for 12 hours to inhibit autophagic flux. Scale bar: 20 µm.

To clarify how RNF138 promotes β-catenin nuclear localization, we examined the RNF138 interactome and identified adenomatous polyposis coli (APC) as a high-confidence interacting partner. Co-IP analysis confirmed the interaction between RNF138 and APC (Figure 4A). In the nucleus, APC binds to β-catenin, mediating its nuclear export and subsequent degradation 39. Based on these findings, we assessed APC expression under various conditions, depending on RNF138 expression levels. Downregulation of RNF138 in C2C12 myoblasts increased APC levels, which correlated with decreased β-catenin levels (Figure 6A-C). In contrast, RNF138 overexpression significantly reduced APC expression (Figure 4F and Figure 6D-E) and its nuclear localization (Figure 6F-H).

To further explore the regulatory interplay among RNF138, APC, and β-catenin, we performed *in vitro* binding assays. While RNF138 directly interacts with β-catenin, no direct interaction between RNF138 and APC was detected in a cell-free system, even in the presence of β-catenin (Figure 6I). Additionally, Co-IP experiments demonstrated that RNF138 minimally affects the APC-β-catenin interaction, suggesting that RNF138 does not regulate β-catenin levels by disrupting APC's binding to β-catenin (Figure 6J).

We next investigated the mechanism by which RNF138 influences APC degradation to stabilize β-catenin. Proteasome inhibition with MG132 did not restore APC levels reduced by RNF138 overexpression, while lysosomal inhibition with bafilomycin A1 (BafA1) preserved APC levels (Figure 6K-L), suggesting that RNF138 may play a role in promoting APC degradation via the autophagy-lysosome pathway, though further studies are needed to fully establish this mechanism in the context of myogenesis 40.

To further investigate the involvement of autophagy in this process, we analyzed the role of autophagy adaptors in APC regulation. Co-IP experiments showed that RNF138 overexpression enhances the interaction between APC and p62, a key autophagy receptor involved in selective cargo degradation (Figure 6J). Moreover, RNF138 was found to interact with both p62 and APC, suggesting a potential role for RNF138 in recruiting APC to p62-mediated selective autophagy 41 (Figure 6M and Figure 4A). Supporting this, IF analysis showed limited colocalization of RNF138 with LC3, an interaction partner of p62 in autophagic flux, under basal conditions. However, treatment with BafA1, which blocks autophagic flux and results in the accumulation of the p62-LC3 complex, significantly increased RNF138-LC3 colocalization (Figure 6N). While these findings indicate that RNF138 may promote APC degradation via p62-mediated autophagy under specific conditions, further investigation is required to establish the direct impact of autophagy on myogenesis.

These results indicate that RNF138 stabilizes β-catenin by promoting APC degradation via the autophagy-lysosome pathway, rather than interfering with the APC-β-catenin binding. This mechanism ensures a reduction in APC levels, allowing β-catenin to accumulate in the nucleus where it facilitates gene regulation during myoblast differentiation. Notably, the specificity of RNF138's role in targeting APC for lysosomal degradation highlights a distinct regulatory axis within the Wnt/β-catenin pathway. This provides a deeper understanding of how RNF138 influences β-catenin stability, beyond transcriptional control, and underscores its importance in maintaining the balance between β-catenin activity and APC-mediated regulation critical for proper myogenic progression.

### RNF138 promotes β-catenin stabilization through ZNF1-mediated RNF138 nuclear localization and APC degradation

**Figure 7:**
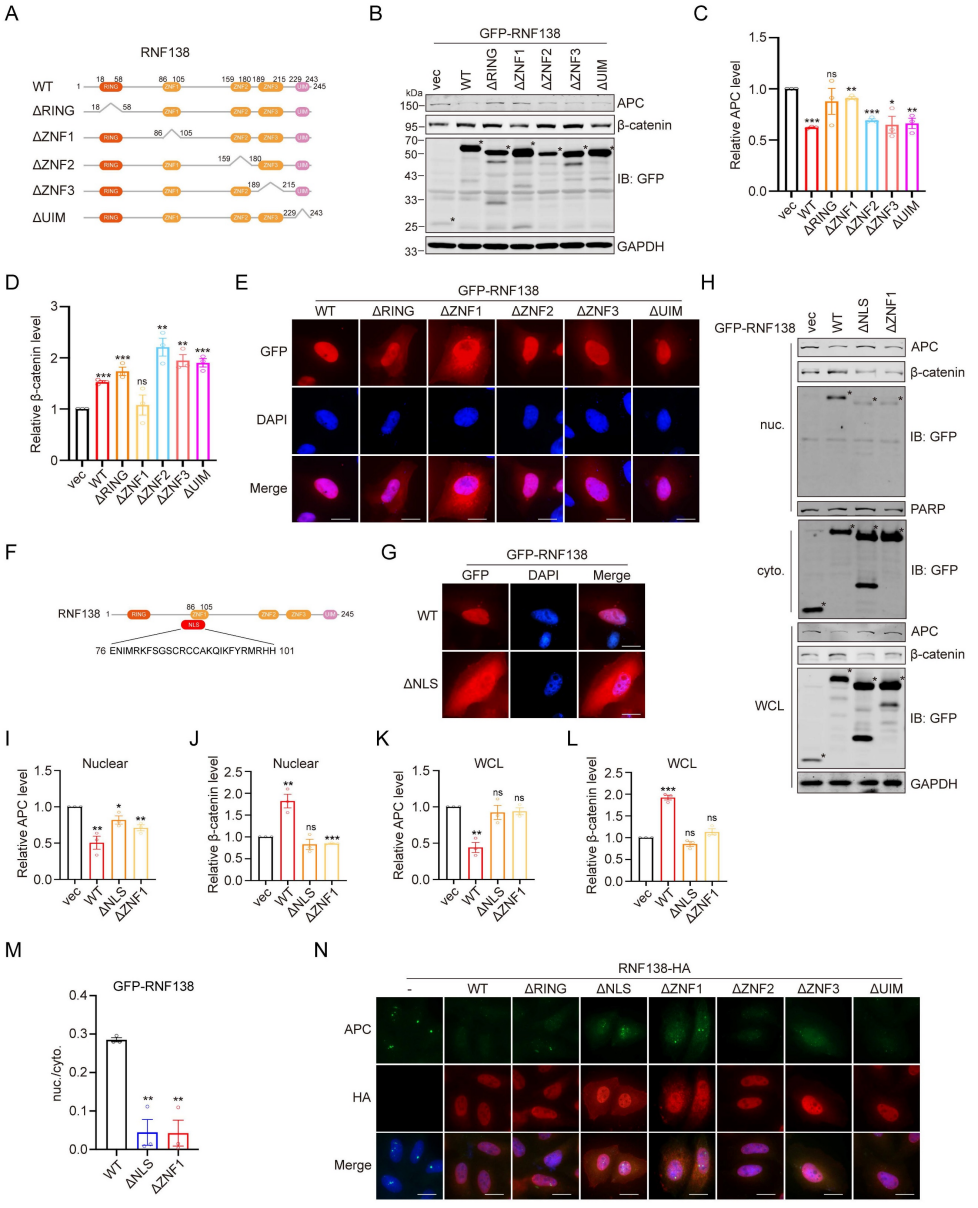
**The effect of RNF138 on β-catenin is dependent on its nuclear localization.** (A) Schematic representation of RNF138 conserved domains and truncation mutants. (B-D) Western blot analysis of β-catenin and APC levels in HEK-293T cells transfected with GFP-tagged RNF138 wild-type (WT) or truncation mutants. (B) Representative Western blot showing the expression of β-catenin and APC, with bands marked by an asterisk (*) indicating target-specific staining. (C) Quantification of relative APC levels, and (D) quantification of relative β-catenin levels, derived from the Western blot analysis shown in Panel (B). Data are presented as mean ± SEM (n = 3). Statistical significance was determined using Student's *t*-test (*p < 0.05, **p < 0.01, ***p < 0.001, ns: not significant). (E) Immunofluorescence (IF) staining of HeLa cells expressing GFP-tagged RNF138 WT or truncation mutants. Nuclei were counterstained with DAPI. Scale bar: 20 μm. (F) Sequence alignment of RNF138 indicating the overlap between ZNF1 and the predicted nuclear localization sequence (NLS) (residues 76-101). (G) IF staining of HeLa cells expressing GFP-tagged RNF138 WT or ΔNLS mutant. Nuclei were counterstained with DAPI. Scale bar: 20 μm. (H) Western blot analysis of nuclear and cytoplasmic fractions of HEK293T cells expressing GFP-tagged RNF138 WT, ΔNLS, or ΔZNF1 mutants. PARP and GAPDH served as nuclear and cytoplasmic markers, respectively. Whole-cell lysates (WCL) were also analyzed. Bands marked with an asterisk (*) indicate target-specific staining. (I-L) Quantification of the relative levels of APC and β-catenin in the nuclear and WCL fractions from the Western blot analysis in Panel (H). Data are presented as mean ± SEM (n = 3). Statistical significance was determined using Student's *t*-test (*p < 0.05, **p < 0.01, ***p < 0.001, ns: not significant). (M) Quantification of the nuclear-to-cytoplasmic ratio of GFP-RNF138, derived from the results shown in Panel (H). Data are presented as mean ± SEM from three independent experiments. Statistical significance was determined by Student's *t*-test, comparing ΔNLS or ΔZNF1 to WT (**p < 0.01). (N) IF staining of HeLa cells expressing HA-tagged RNF138 WT or mutants. Nuclei were counterstained with DAPI. Scale bar: 20 μm.

To elucidate the structural basis by which RNF138 stabilizes β-catenin, we generated a series of truncation mutants of RNF138, with the deletion of the RING, ZNF1, ZNF2, ZNF3, or UIM domain (Figure 7A). Western blot analysis revealed that deletion of the ZNF1 domain (ΔZNF1) abolished RNF138-mediated β-catenin stabilization and led to elevated APC levels, emphasizing the critical role of ZNF1 in facilitating APC degradation (Figure 7B-D). To assess whether ZNF1 contributes to RNF138's nuclear localization, we performed IF analysis (Figure 7E), which showed that ZNF1 deletion significantly reduced RNF138 nuclear localization. Subsequently, we investigated the nuclear localization sequence (NLS) within RNF138, as bioinformatic predictions indicated substantial overlap between the NLS and ZNF1 (Figure 7F). Confirming this, IF analysis of RNF138 mutants lacking the predicted NLS showed markedly decreased nuclear localization (Figure 7G).

In subsequent experiments, we explored the impact of impairing RNF138's nuclear localization on β-catenin stabilization. Overexpression of the RNF138 NLS deletion mutant led to reductions in both total and nuclear β-catenin levels, with a concurrent increase in APC expression (Figure 7H-L). These data indicate that RNF138's nuclear localization is essential for its role in stabilizing β-catenin and modulating APC levels. Nuclear-cytoplasmic fractionation experiments further confirmed that both the NLS and ZNF1 domains are essential for effective RNF138 translocation to the nucleus, as evidenced by the nuclear-to-cytoplasmic ratio of RNF138 (Figure 7H and 7M). Finally, IF analysis demonstrated that the loss of RNF138 nuclear localization also impaired its ability to decrease nuclear APC levels, as observed in the mutant (Figure 7N).

In summary, these findings demonstrate that RNF138's nuclear localization, driven by its ZNF1 domain or NLS, is necessary for its role in promoting APC degradation and stabilizing β-catenin.

## Discussion

In this study, we demonstrated that RNF138 regulates β-catenin stability by reducing APC levels, rather than disrupting the APC-β-catenin interaction. APC is a key negative regulator of the Wnt/β-catenin pathway, responsible for promoting β-catenin nuclear export and cytoplasmic degradation through proteasomes. This nuclear export function of APC ensures proper control over β-catenin-mediated gene activation. By targeting APC for degradation via the autophagy-lysosome pathway, RNF138 facilitates the accumulation of β-catenin in the nucleus, thereby activating downstream transcriptional programs essential for myogenic differentiation.

Wnt/β-catenin signaling plays crucial roles in diverse biological processes, including cell fate determination, proliferation, and tissue regeneration. The ability of RNF138 to modulate APC stability positions it as a potential regulator of these processes, particularly in tissues where APC expression is tightly controlled. Additionally, while our study identifies lysosomal degradation of APC as the primary mechanism for RNF138, other E3 ligases such as MKRN1 have been reported to target APC for proteasomal degradation 42. Data from tissue correlation analyses further support this distinction (Figure S7). RNF138 and MKRN1 expression levels showed positive correlations across various tissues, including skeletal muscle, pancreas, and kidney cortex, suggesting a potential interplay or compensatory relationship between these two E3 ligases. Proteasomal degradation is often considered a rapid-response mechanism for eliminating short-lived or misfolded proteins, making MKRN1 more likely to dominate APC regulation in tissues with higher metabolic activity and proteasomal activity. In contrast, RNF138 may take precedence in contexts where autophagy-lysosome pathways are more active, such as during cellular stress or in long-term protein turnover processes. This complementary or context-dependent regulation could explain how APC stability and β-catenin signaling are fine-tuned under diverse physiological conditions.

Furthermore, APC's role in shuttling β-catenin out of the nucleus introduces an additional layer of complexity. The differential regulation of APC by RNF138 and MKRN1 may not only affect β-catenin stabilization but also its subcellular localization, potentially influencing transcriptional activity. These observations underscore the importance of tissue-specific regulatory mechanisms in modulating Wnt/β-catenin signaling.

Future studies should delve deeper into the upstream signals that modulate RNF138 activity in various tissues and contexts. Additionally, elucidating the precise molecular interplay between RNF138 and MKRN1, and their differential contributions to APC regulation, will provide further insights into the fine-tuning of Wnt signaling and its physiological relevance.

### Limitations of the study

Despite the significant insights provided in this study regarding RNF138's regulation of APC stability and its involvement in Wnt/β-catenin signaling, several key limitations remain, particularly with respect to the detailed mechanisms of degradation and the regulation of APC and RNF138.

### Unresolved degradation mechanisms

RNF138 targets APC for degradation via the autophagy-lysosome pathway, yet the detailed molecular mechanisms remain to be elucidated. Specifically, the steps involved in the lysosmal degradation of APC by RNF138 are not fully delineated. Future work should aim to identify the specific autophagy-related proteins or complexes involved in this process, as well as the exact molecular interactions between RNF138 and the components of the autophagic machinery.

### Proteasomal vs. lysosomal degradation

While this study focuses on RNF138's role in APC degradation through the autophagy-lysosome pathway, it is known that the proteasome also plays an essential role in APC degradation. The interplay between these two degradation pathways in controlling APC protein levels is still not fully determined. Future work on this topic may help to generate a more complete picture of the degradation mechanisms of APC, particularly those that are tissue-specific.

### Regulation of RNF138 activity

The mechanisms regulating RNF138 itself, such as post-transcriptional modifications or interactions with other proteins, are not fully understood. Identifying how RNF138 is activated in specific physiological conditions will be crucial for understanding its role in regulating APC stability.

### The link between DNA damage repair and myoblast differentiation

RNF138 is well known for its essential role in DNA damage repair. Interestingly, stem cells often exhibit a DNA damage-like phenotype during differentiation, likely due to rapid cell division or the transition of stem cells from a quiescent to a proliferative state 25, 43. The function of RNF138, as uncovered in this study, suggests that it could be involved in bridging DDR and stem cell differentiation, which is an important topic that awaits future investigation.

## Summary

In summary, this study establishes RNF138 as a crucial regulator of β-catenin by targeting APC for degradation via the autophagy-lysosome pathway, thereby providing a novel mechanism for modulating Wnt/β-catenin signaling. This insight suggests that RNF138 could serve as a potential therapeutic target in diseases where Wnt signaling is dysregulated, such as cancers and degenerative disorders. Future studies should focus on elucidating the molecular details of RNF138's interaction with APC and exploring its broader implications in tissue-specific signaling contexts.

## Materials and methods

### Cell cultures

HEK-293T, A549, HeLa, U2OS, and C2C12 cells were cultured in DMEM (Cat# 11965, Gibco, USA) supplemented with 10% fetal bovine serum (Cat# A5670501, Gibco, USA) and 1% penicillin/streptomycin (Cat# 15140122, Gibco, USA). C2C12 myoblasts were differentiated in DMEM with 2% horse serum (Cat# BL209A, Biosharp, China). Cells were maintained at 37°C in a 5% CO_2_ incubator and passaged at 80% confluence using 0.25% trypsin-EDTA (Cat# 25200072, Gibco, USA).

### Mice

All animal experiments were conducted using C57BL/6JGpt mice (GemPharmatech, China), housed under specific pathogen-free (SPF) conditions during the study. The *Rnf138*-knockout (KO) mice model was generated using CRISPR/Cas9 technology. The targeted knockout region was designed based on the *Rnf138*-203 transcript (ENSMUST00000234107.1), focusing on exon 3, which contains a 166 bp coding sequence. Deletion of this region disrupts RNF138 protein function.

For model generation, two sgRNAs targeting the intronic regions flanking exon 3 of *Rnf138* were designed and co-injected with the CRISPR/Cas9 system into C57BL/6JGpt zygotes. The edited zygotes were transferred into surrogate mothers, and the resulting F0 mice were genotyped and confirmed by PCR and sequencing. F0 mice carrying the desired mutation were bred with wild-type C57BL/6JGpt mice to produce an F1 generation of heterozygous *Rnf138*-KO mice. Homozygous *Rnf138*-KO mice were subsequently generated by intercrossing F1 heterozygous mice, with offspring genotypes confirmed via PCR.

All experimental procedures were reviewed and approved by the Institutional Animal Care and Use Committee (IACUC), Sun Yat-sen University (Approval No. SYSU-IACUC-2024-001487).

### CTX-induced muscle injury

Cardiotoxin (CTX) (Cat# HY-P1902, MedChemExpress, USA) was dissolved in sterile saline to prepare a 10 mM working solution. Eight-week-old C57BL/6JGpt mice were anesthetized with isoflurane (Cat# R510-22-10, RWD Life Science, China), and the tibialis anterior (TA) muscles were exposed by shaving the anterior hind limb and disinfecting the area with 75% ethanol. With a 26-gauge needle, 50 μL of the 10 mM CTX solution was injected intramuscularly into the TA muscle at a depth of 2-3 mm with a 10°-20° angle to minimize leakage. After the procedure, mice were placed on a 37°C heating pad until fully recovered. Tissue samples were collected at designated days post-injection (dpi) for downstream analyses.

### Lentiviral-mediated gene knockdown and overexpression

Gene knockdown cell lines were established using the pLKO.1-puro lentiviral system (Cat# 10878, Addgene, USA), while gene overexpression cell lines were generated using the pLV-EF1a-IRES-Blast lentiviral system (Cat# 85133, Addgene, USA). Lentiviral particles were produced by co-transfecting the expression plasmid with psPAX2 (Cat# 12260, Addgene, USA) and pMD2.G (Cat# 12259, Addgene, USA) into HEK-293T cells using Liposomal Transfection Reagent (Cat# HB220930, Yeasen Biotechnology, China). The resulting lentiviral supernatants were harvested, filtered, and used to infect target cell lines in the presence of 8 μg/mL polybrene (Cat# H9268, Sigma-Aldrich, USA). After selection with appropriate antibiotics, stable cell lines were established. Gene knockdown and overexpression efficiencies were confirmed by Western blotting (WB) and quantitative real-time PCR (qRT-PCR), respectively.

### Transient protein expression

Transient protein expression was achieved using the pcDNA3.1 (+) expression vector (Cat# V79020, Invitrogen, USA). The coding DNA sequence (CDS) of the target gene was inserted into the pcDNA3.1(+) vector. For HEK-293T, A549, and HeLa cells, transfections were performed using Liposomal Transfection Reagent (Cat# HB220930, Yeasen Biotechnology, China) according to the manufacturer's protocol. For C2C12 myoblasts, transfections were carried out using LipoMax (Cat# 32105, Sudgen, China) following the manufacturer's instructions. After 24-48 hours of transfection, cells were harvested for downstream analyses, including Western blotting (WB), quantitative real-time PCR (qRT-PCR), immunoprecipitation (IP), and immunofluorescence (IF).

### RNA isolation and quantitative real-time PCR

Total RNA was extracted from cell samples using the VeZol reagent (Cat# R411-01, Vazyme, China) according to the manufacturer's instructions. RNA purity and concentration were determined using a NanoDrop spectrophotometer (Thermo Fisher Scientific, USA). RNA was reverse-transcribed into cDNA using the HiScript II Q RT SuperMix (Cat# R223-01, Vazyme, China).

Quantitative real-time PCR (qRT-PCR) was performed using the ChamQ Universal SYBR qPCR Master Mix (Cat# Q711-02, Vazyme, China) on a StepOnePlus Real-Time PCR System (Applied Biosystems, USA). The relative expression levels of target genes were calculated using the 2^-ΔΔCt method, with GAPDH as the internal control.

### RNA-Seq and analysis

mRNA was enriched from total RNA using Oligo dT magnetic beads. Library quality was assessed using a Qubit 2.0 Fluorometer (Thermo Fisher Scientific, USA) for initial quantification, followed by an Agilent 2100 Bioanalyzer (Agilent Technologies, USA) for insert size analysis. The library concentration was validated by qRT-PCR to ensure an effective concentration greater than 1.5 nM. After pooling, libraries were subjected to Illumina sequencing using the Sequencing by Synthesis (SBS) method. This entire process was performed by Shi-Biotech (Wuhan, China).

### Chromatin immunoprecipitation (ChIP)

ChIP was performed using the SimpleChIP Enzymatic Chromatin IP Kit (Cat# 9003, CST, USA) with HEK-293T cells transfected with HA-RNF138. Cells were crosslinked with 1% formaldehyde, quenched with glycine, and washed with ice-cold PBS. Chromatin was prepared by digesting nuclei with micrococcal nuclease (Cat# 10011, CST, USA) and sonicating to release fragments of 150-900 bp.

For immunoprecipitation, 5 μg of chromatin was incubated with anti-HA-tag (targeting RNF138) (Cat# 3724, CST, USA), anti-Histone H3 (positive control) (Cat# 4620, CST, USA), or normal rabbit IgG (negative control) (Cat# 2729, CST, USA) at 4°C, followed by capture with Protein G magnetic beads (Cat# 9006, CST, USA). Immunoprecipitated chromatin was washed sequentially with low-salt and high-salt buffers, eluted, and reverse crosslinked. Purified DNA was analyzed by quantitative real-time PCR (qRT-PCR) using primers for *RPL30*, *GAPDH*, *RPL13A*, and *MYC* as controls.

### Protein extraction and western blotting

Protein samples were extracted using RIPA buffer (Cat# CW2333S, CWBIO, China) supplemented with protease inhibitors (Cat# CW2200S, CWBIO, China). Protein concentrations were determined using a BCA protein assay kit (Cat# 23225, Thermo Fisher Scientific, USA). Protein (10-20 µg per sample) was separated by SDS-PAGE and transferred onto 0.45 μm nitrocellulose (NC) membranes (Cat# HATF00010, Sigma-Aldrich, USA).

The membranes were blocked with 5% non-fat milk in TBS-T for 1 hour at room temperature and incubated overnight at 4°C with the indicated primary antibodies:

anti-RNF138 (Cat# A10304, ABclonal, China)

anti-MyHC (Cat# sc-376157, Santa Cruz, USA)

anti-eMyHC (Cat# sc-53091, Santa Cruz, USA)

anti-Myogenin (Cat# ab1835, Abcam, UK)

anti-APC (Cat# ab40778, Abcam, UK)

anti-β-catenin (Cat# 51067-2-AP, Proteintech, China)

anti-p62 (Cat# H00008878-M01, Novus Biologicals, USA)

anti-Phospho-Histone H2A.X (Ser139) (Cat# 9718, CST, USA)

anti-Histone H3 (Cat# sc-517576, Santa Cruz, USA)

anti-GAPDH (Cat# 10494-1-AP, Proteintech, China)

anti-β-actin (Cat# 60008-1-Ig, Proteintech, China)

anti-Tubulin (Cat# 11224-1-AP, Proteintech, China)

anti-PARP (Cat# 9542, CST, USA)

anti-HA-Tag (Cat# 3724, CST, USA)

anti-GFP-tag (Cat# ABM40124, Abbkine, China)

anti-His-tag (Cat# 66005-1-Ig, Proteintech, China)

anti-GST (Cat# sc-138, Santa Cruz, USA)

anti-Flag (Cat# F1804, Sigma-Aldrich, USA)

After washing three times with TBS-T, membranes were incubated with fluorescent secondary antibodies:

IRDye® 800CW Goat anti-Rabbit IgG Secondary Antibody (Cat# 926-32211, Li-COR Biosciences, USA)

IRDye® 680CW Goat anti-Mouse IgG Secondary Antibody (Cat# 926-68070, Li-COR Biosciences, USA)

Incubation was performed for 1 hour at room temperature. The membranes were then washed three or more times with TBS-T. Protein bands were visualized using a Li-COR Odyssey infrared imaging system (Li-COR Biosciences, USA) at the appropriate wavelengths for each antibody.

### Hematoxylin and Eosin (H&E) staining

TA muscle tissues were fixed in 4% paraformaldehyde at 4°C for 48 hours, embedded in paraffin, and sectioned at 5 µm. Sections were deparaffinized, rehydrated, stained with hematoxylin (2 min), differentiated in 0.5% acid alcohol, blued in 0.5% ammonia water, counterstained with eosin (30 s), and dehydrated. Finally, sections were cleared with xylene and mounted.

### Immunohistochemistry (IHC)

Paraffin sections were deparaffinized, rehydrated, and subjected to antigen retrieval with citrate buffer (pH 6.0) for 20 minutes. Endogenous peroxidase activity was blocked with 3% hydrogen peroxide, followed by overnight incubation with anti-RNF138 (Cat# A10304, ABclonal, China) primary antibodies at 4°C. After washing with PBS, sections were incubated with HRP-conjugated secondary antibodies for 1 hour at room temperature, developed using DAB (Cat# abs9210-1kit, absin, China), and counterstained with hematoxylin. Sections were then dehydrated, cleared, mounted, and examined under a light microscope.

### Immunofluorescence (IF)

Cells were seeded onto coverslips and incubated as required. After washing with PBS, cells were fixed with paraformaldehyde for 15 minutes and washed again with PBS. Permeabilization was performed using 0.1% Triton X-100 for 10 minutes, followed by washes with PBS and PBST. Cells were blocked with 5% goat serum for 1 hour at room temperature. Primary antibodies against HA (Cat# 3724, CST, USA), Flag (Cat# F1804, Sigma-Aldrich, USA), APC (Cat# ab40778, Abcam, UK), MyHC (Cat# sc-376157, Santa Cruz, USA), and β-catenin (Cat# 51067-2-AP, Proteintech, China) were incubated overnight at 4°C. After washing, cells were incubated with anti-Rabbit secondary antibody (Cat# A-11008, Invitrogen, USA) or anti-Mouse secondary antibody (Cat# A-11005, Invitrogen, USA) for 1 hour at room temperature. For nuclear staining, DAPI (1 ng/mL) was added, and cells were washed with PBS. Finally, the coverslips were mounted, and fluorescence images were captured using either a Zeiss Axio Observer 7 microscope or a Zeiss LSM900 confocal microscope (Germany), as required by the experimental setup.

### Co-immunoprecipitation (Co-IP)

HEK-293T cells transfected with GFP-RNF138 or GFP control plasmids were harvested by scraping and pelleted at 800 x g for 5 minutes at 4°C. The cells were lysed in IP lysis buffer (30 mM Tris, pH 7.5, 150 mM NaCl, 10% glycerol, 1% Triton X-100, 0.5 mM EDTA, 10 mM NaF, 100 µM sodium orthovanadate, and 200 µM PMSF) supplemented with a protease inhibitor cocktail (Cat# CW2200S, CWBIO, China). Approximately 1-2 mg of total protein was used for each Co-IP reaction. Lysis was performed on ice for 1 hour, and the lysates were cleared by centrifugation at 12,000 x g for 10 minutes at 4°C.

For immunoprecipitation, 10 µL of anti-GFP nanobody agarose beads (Cat# KTSM1301, AlpaLifeBio, China) were pre-washed three times with PBS-T and then incubated with the cleared lysates overnight at 4°C with gentle rotation. After incubation, the beads were washed three times with PBS-T to minimize nonspecific binding. The immunoprecipitated complexes were eluted by boiling the beads in 2x SDS sample buffer at 95°C for 10 minutes. The eluted proteins were analyzed using Western blotting as described.

### Recombinant protein purification and GST-pull-down

Recombinant proteins were expressed in *E. coli* BL21 (DE3) cells using the pGEX-6P-1 vector for GST-tagged proteins and the pET-28a vector for His-tagged proteins. Protein expression was induced by 0.5 mM IPTG at 16°C for 12-16 hours. After harvesting, cells were lysed in pull-down buffer (25 mM Tris, 300 mM NaCl, 2 mM DTT, 2% [v/v] glycerol, 0.1% Tween-20, pH 8.0) containing protease inhibitors by sonication. The lysates were clarified by centrifugation at 15,000 x g for 20 minutes.

For the GST pull-down assay, purified recombinant proteins were incubated together in the pull-down buffer overnight at 4°C with gentle rotation to allow for protein-protein interactions. Pre-washed GST beads were then added to the mixture and incubated for 2 hours at 4°C. After incubation, the beads were washed three times with pull-down buffer, and the bound proteins were eluted with SDS sample buffer. The eluted proteins were analyzed by SDS-PAGE and Western blotting.

### Subcellular protein fractionation

Subcellular fractionation was performed using the Subcellular Protein Fractionation Kit for Cultured Cells (Cat# 78840, Thermo Fisher Scientific, USA) following the manufacturer's instructions. This protocol yielded cytoplasmic, membrane, nuclear, and chromatin-bound protein fractions for downstream analyses.

### Statistical analysis

Statistical analyses were carried out using GraphPad Prism (version 10.2.0). Data are expressed as mean ± SEM from at least three independent experiments. Two-group comparisons were performed with an unpaired Student's *t*-test. A *p*-value of less than 0.05 was considered statistically significant, and detailed p-values are provided in the figure legends.

## Supplementary Material

Supplementary figures and tables.

## Abbreviations

MRFs: myogenic regulatory factors
bHLH: basic helix-loop-helix
APC: adenomatous polyposis coli
GSK-3β: glycogen synthase kinase-3β
CK1α: casein kinase 1α
GEO: gene expression omnibus
MyHC: myosin heavy chain
SCR: scrambled control
IF: immunofluorescence
DEGs: differentially expressed genes
GM: growth medium
DMdx: differentiation medium day x
GO: gene ontology
Il6: interleukin-6
Tgfbr3: transforming growth factor beta receptor 3
TA: tibialis anterior
CTX: cardiotoxin
H&E: hematoxylin and eosin
IHC: immunohistochemistry
eMyHC: embryonic myosin heavy chain
dpi: days post-injection
WT: wild-type
KO: knockout
CtIP: CtBP-interacting protein
dsDNA: double-stranded DNA
ssDNA: single-stranded DNA
ChIP: chromatin immunoprecipitation
GFP: green fluorescent protein
Co-IP: co-immunoprecipitation
MS: mass spectrometry
As: arsenic trioxide
Eto: etoposide
CE: cytoplasmic extract
ME: membrane extract
NE: nuclear extract
CB: chromatin-bound fraction
GST: glutathione-S-transferase
BafA1: bafilomycin A1
NLS: nuclear localization sequence
DDR: DNA damage repair
