# Supplementary material for: RNF138 regulates skeletal muscle differentiation via the Wnt/β-catenin signaling pathway
Source: Theranostics. 2025 Mar 18;15(10):4446–64. doi: 10.7150/thno.110925 (PMC11984406; doi:10.7150/thno.110925)

Supplementary Figure 1. Construction of *Rnf138*-knockdown (*Rnf138*-KD) C2C12 cell lines.

(A) Western blot analysis of RNF138 protein levels in stable *Rnf138*-KD C2C12 myoblast cell lines. Cells were infected with lentivirus-mediated shRNA targeting *Rnf138* (shRnf138 #1, #2, and #3) or a scrambled control (SCR).

(B) Quantification of *Rnf138* mRNA levels in stable *Rnf138*-KD and SCR C2C12 cell lines. Relative mRNA expression of *Rnf138* was measured by qPCR. Data are normalized to the values in the SCR and presented as the mean ± SEM and statistical significance was determined using Student's *t*-test, comparing each *Rnf138*-KD group to the SCR group (***$p < 0.001$).

Supplementary Figure 2. Clustering analysis of differentially expressed genes (DEGs) following *Rnf138*- knockdown (*Rnf138*-KD) in proliferating C2C12 myoblasts.

(A) Venn diagram showing the overlap and unique gene expression profiles between *Rnf138*-KD and control (SCR) proliferating C2C12 myoblasts. Numbers indicate unique and shared genes between the two groups.

(B) Volcano plot illustrating the distribution of DEGs between *Rnf138*-KD and control groups. DEGs with statistical significance (adjusted p-value < 0.05) and log2(Fold Change) > 1 are highlighted in green (downregulated) and red (upregulated).

(C) Heatmap of DEGs identified between RNF138 knockdown and control groups, showing hierarchical clustering of samples and genes. Red represents upregulated genes, and green represents downregulated genes.

(D) Gene ontology (GO) biological process (BP) enrichment analysis of significant DEGs, highlighting the top 10 enriched terms.

(E-H) Heatmaps showing the expression levels of key genes within specific GO categories: (E) "muscle organ development," (F) "muscle cell development," (G) "muscle-skeletal system development," and (H) "muscle cell differentiation."

Supplementary Figure 3. GO analysis RNF138 interactome based on IP-MS.

Co-immunoprecipitation followed by mass spectrometry (Co-IP/MS) was performed in HEK293 cells overexpressing GFP-RNF138, with GFP as a control. The identified RNF138 interactome was analyzed through Gene Ontology (GO) enrichment. GO terms were categorized into biological processes (A), molecular functions (B), and cellular components

(C), and significant enrichments were determined based on -log10(p-value) and percentage of proteins.

Supplementary Figure 4. *Ctnnb1*-knockdown (*Ctnnb1*-KD) impairs C2C12 myoblasts differentiation.

(A) Western blot analysis of β-catenin protein levels in C2C12 cells transfected with scrambled control (SCR) or shCtnnb1 constructs (#1, #2, #3).

(B) Relative mRNA levels of *Ctnnb1* in C2C12 cells were assessed by qRT-PCR. Data are normalized to the values in the SCR and presented as mean ± SEM and statistical significance was determined using Student's *t*-test, comparing each shCtnnb1 group to the SCR group (*$p < 0.05$, **$p < 0.01$).

(C) Western blot analysis of MyHC, RNF138, and β-catenin levels in *Ctnnb1*-KD (shCtnnb1#1 and shCtnnb1#3) and SCR cells after 5 days of differentiation (DMd5).

(D) Representative immunofluorescence images of MyHC (red) and nuclei (DAPI, blue) in differentiated C2C12 myoblasts (DMd5) with SCR or *Ctnnb1*-KD (shCtnnb1#1 and shCtnnb1#3). Scale bar: 100 μm.

(E) Quantification of MyHC-positive cells (%) from (D). Data are presented as mean ± SEM from three independent fields per group. Statistical significance was determined using Student's *t*-test, comparing each *Rnf138*-KD group to the SCR group (***$p < 0.001$).

(F) Relative mRNA expression levels of myogenic markers *Myod1*, *Myogenin* (*Myog*), *Myomaker*, and *Myh2b* in SCR and *Ctnnb1*-KD cells after 2 days of differentiation (DMd2). Data are normalized to the values in the SCR group and presented as mean ± SEM from three independent experiments. Statistical analysis was performed using Student's *t*-test, comparing each *Ctnnb1*-KD group to the SCR group (***$p < 0.001$).

(G) Relative mRNA levels of *Myod1*, *Myog*, *Myomaker*, and *Myh2b* in C2C12 myoblasts treated with DMSO (control) or XAV939 (Wnt/β-catenin inhibitor) after 2 days of differentiation (DMd2). Data are normalized to the values in the SCR group and presented as mean ± SEM from three independent experiments. Statistical analysis was performed using Student's *t*-test, comparing the XAV929 group to the DMSO group (*$p < 0.05$, **$p < 0.01$, ***$p < 0.001$).

Supplementary Figure 5. β-catenin is positively correlated with RNF138.

(A) Western blot analysis of β-catenin levels in A549 cells transduced with scrambled control (SCR) or RNF138 knockdown constructs (shRNF138 #1 and #2).

(B) Time-course analysis of β-catenin protein levels in A549 cells overexpressing GFP-RNF138 for 24, 36, and 48 hours.

(C) Relative mRNA levels of *CTNNB1* in A549 cells transfected with GFP or GFP-RNF138. Data are normalized to the values in the GFP group and presented as mean ± SEM from three independent experiments. Statistical analysis was performed using Student's *t*-test, comparing the GFP-RNF138 group to the GFP group (ns: not significant).

(D, E) Western blot analysis of β-catenin, RNF138, and γ-H2A.X (Ser139) levels in HEK293T, U2OS, and C2C12 cells treated with arsenate (As) (D) or etoposide (Eto) (E).

Supplementary Figure 6. GO analysis of DEGs related to the canonical Wnt signaling pathway upon *Rnf138*-knockdown.

Heatmap of differentially expressed genes (DEGs) related to the canonical Wnt signaling pathway in differentiating (A) and proliferating (B) myoblasts upon *Rnf138*-knockdown.

Supplementary Figure 7. Correlation analysis of RNF138 and MKRN1 mRNA expression in various human tissues.

Scatter plots showing the correlation between RNF138 and MKRN1 mRNA expression levels across various human tissues. Data were generated using the online tool GEPIA (http://gepia.cancer-pku.cn/) on November 24, 2024. Pearson correlation coefficients (R) and *p*-values are shown in the corresponding figures. All analyses were conducted entirely by the GEPIA platform without manual intervention.

Table S1. The sequences of primers used in this study.

Name	Sequence (5' ~ 3')
**Primers used for generating constructs (Mus musculus)**	
Rnf138-F	ATGTCCGAGGAACTTTCGG
Rnf138-R	TCACATGTTTACTTGAAAAGACTCTT
**Primers used for generating constructs (Homo sapiens)**	
RNF138-F	ATGGCCGAGGACCTCTC
RNF138-R	TCAGATGTTTACTTGAAAAGATTCTT
β-catenin-F	ATGGCTACTCAAGCTGATTTG
β-catenin-R	TTACAGGTCAGTATCAAACCAGG
APC-Nter-F	CAGTCTGCTGGATTTGGTTCTAGGGTG
APC-Nter-R	TTAAACAGATGTCACAAGGTAAGACCC
**Primers used to generate deletion mutants of RNF138 for functional domain studies (Homo sapiens)**	
RNF138-ΔRING-F	CGAAGATGATTTCTACGGAAATGTGACTAGAAGAGAGAGAGCATGTC
RNF138-ΔRING-R	TTCTAGTCACATTTCCGTAGAAATCATCTTCGGTGTAGGACGTGGC
RNF138-ΔZNF1-F	GAAGTTTTCTGGTAGCAAGAAGTATCAGGATGAATATGGTGTTTC

RNF138-ΔZNF1-R	TTCATCCTGATACTTCTTGCTACCAGAAAACTTCCTCATTATATTTTC
RNF138-ΔZNF2-F	GTCATCCTACTTTTAAGCTATTTCAGATAGTTCCTGTGACATG
RNF138-ΔZNF2-R	CAGGAACTATCTGAAATAGCTTAAAAGTAGGATGACCAGAAGAAC
RNF138-ΔZNF3-F	GATAGTTCCTGTGACACAATTTGATTATGGAGAATTTGTGAATC
RNF138-ΔZNF3-R	TTCTCCATAATCAAATTGTGTCACAGGAACTATCTGAAATAGG
RNF138-ΔUIM-F	GAATCTTCAGCTAGATAACATCTGAGAATTCTGCAGATATC
RNF138-ΔUIM-R	CAGAATTCTCAGATGTTATCTAGCTGAAGATTCACAAATTCTC
RNF138-ΔNLS-F	GCCTTAGACCTTGAAAATATATACAAATCTTGTAAGAAGTATCAGGATG
RNF138-ΔNLS-R	GATACTTCTTACAAGATTTGTATATATTTTCAAGGTCTAAGGCCCG
**Primers used for constructing vectors expressing short hairpin RNAs (shRNAs) of various genes (Mus musculus)**	
Rnf138_shRNA#1-F	CCGGCTTCTGTCATTCCAAACTTTACTCGAGTAAAGTTTGGAATGACAGAAGTTTTT
Rnf138_shRNA#1-R	AATTAAAAACTTCTGTCATTCCAAACTTTACTCGAGTAAAGTTTGGAATGACAGAAG
Rnf138_shRNA#2-F	CCGGCTGCATCTGATAACACAGAAACTCGAGTTTCTGTGTTATCAGATGCAGTTTTT
Rnf138_shRNA#2-R	AATTAAAAACTGCATCTGATAACACAGAAACTCGAGTTTCTGTGTTATCAGATGCAG
Rnf138_shRNA#3-F	CCGGCCCTTATGTCAAGAGTCAAATCTCGAGATTTGACTCTTGACATAAGGGTTTTT
Rnf138_shRNA#3-R	AATTAAAAACCCTTATGTCAAGAGTCAAATCTCGAGATTTGACTCTTGACATAAGGG
Ctnnb1_shRNA#1-F	CCGGGCTGATATTGACGGGCAGTATCTCGAGATACTGCCCGTCAATATCAGCTTTTT
Ctnnb1_shRNA#1-R	AATTAAAAAGCTGATATTGACGGGCAGTATCTCGAGATACTGCCCGTCAATATCAGC
Ctnnb1_shRNA#2-F	CCGGCACGCAAGAGCAAGTAGCTGATATTCTCGAGAATATCAGCTACTTGCTCTTGCGTGTTTTT
Ctnnb1_shRNA#2-R	AATTAAAAACACGCAAGAGCAAGTAGCTGATATTCTCGAGAATATCAGCTACTTGCTCTTGCGTG
Ctnnb1_shRNA#3-F	CCGGCCCTCAGATGGTGTCTGCCATTGTACTCGAGTACAATGGCAGACACCATCTGAGGGTTTTT
Ctnnb1_shRNA#3-R	AATTAAAAACCCTCAGATGGTGTCTGCCATTGTACTCGAGTACAATGGCAGACACCATCTGAGGG
**Primers used for constructing vectors expressing short hairpin RNAs (shRNAs) of various genes (Homo sapiens)**	
RNF138_shRNA#1-F	CCGGGCTAGATGAAGAAACCCAATACTCGAGTATTGGGTTTCTTCATCTAGCTTTTT
RNF138_shRNA#1-R	AATTAAAAAGCTAGATGAAGAAACCCAATACTCGAGTATTGGGTTTCTTCATCTAGC
RNF138_shRNA#2-F	CCGGGTCGTGGAAATGTGACTAGAACTCGAGTTCTAGTCACATTTCCACGACTTTTT
RNF138_shRNA#2-R	AATTAAAAAGTCGTGGAAATGTGACTAGAACTCGAGTTCTAGTCACATTTCCACGAC
RNF138_shRNA#3-F	CCGGGCATGAGACATCATTACAAATCTCGAGATTTGTAATGATGTCTCATGCTTTTT
RNF138_shRNA#3-R	AATTAAAAAGCATGAGACATCATTACAAATCTCGAGATTTGTAATGATGTCTCATGC
**Primers used for qRT-PCR (Mus musculus)**	
Rnf138-f	TTCTACTGCCCTGTCTGTCA
Rnf138-r	CGTTCCGGACATGCTCTTTC
Myod1-f	CCACTCCGGGACATAGACTTG
Myod1-r	AAAAGCGCAGGTCTGGTGAG
Myogenin-f	CTACAGGCCTTGCTCAGCTC
Myogenin-r	CACGATGGACGTAAGGGAGT
Mrf4-f	CGGCTGGATCAGCAAGAGAA
Mrf4-r	CCACGTTTGCTCCTCCTTCC
Myomaker-f	ATCGCTACCAAGAGGCGTT
Myomaker-r	CACAGCACAGACAAACCAGG
Myh2b-f	TGATGCAGGCTGAGATCGAGGAG
Myh2b-r	TTGGTGTTGATGAGGCTGGTGTTC
IL-6-f	GAACAACGATGATGCACTTGC
IL-6-r	CTTCATGTACTCCAGGTAGCTATGGT
Fos-f	GTTTCCGGCATCATCTAGGC

Fos-r	GAAGGAGTCAGCTTCAGGGT
Sox8-f	CAGAGCTCAGCAAGACCCTA
Sox8-r	TCACACTCTTCCTTCGCCTT
Notch1-f	ACACCGTGTAAGAATGCTGGA
Notch1-r	GCCTGCTGACATGATTTTCCTG
Ctnnb1-f	CACAACCTTTCTCACCACCG
Ctnnb1-r	TTTCTGCAGTCCACCAGCTA
Gapdh-f	GACGTGCCGCCTGGAGA
Gapdh-r	GAAGAGTGGGAGTTGCTGTTGAA

WANG et al. **Suppl. Fig. 1**

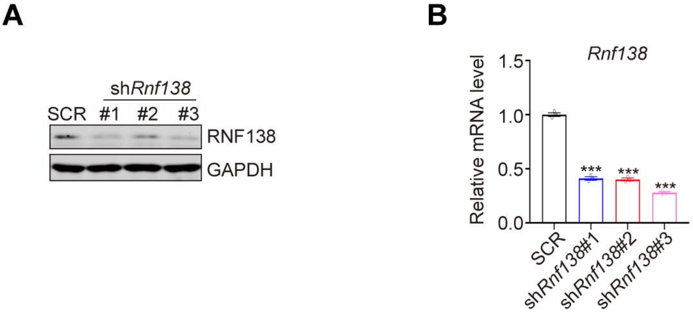

WANG et al. **Suppl. Fig. 2**

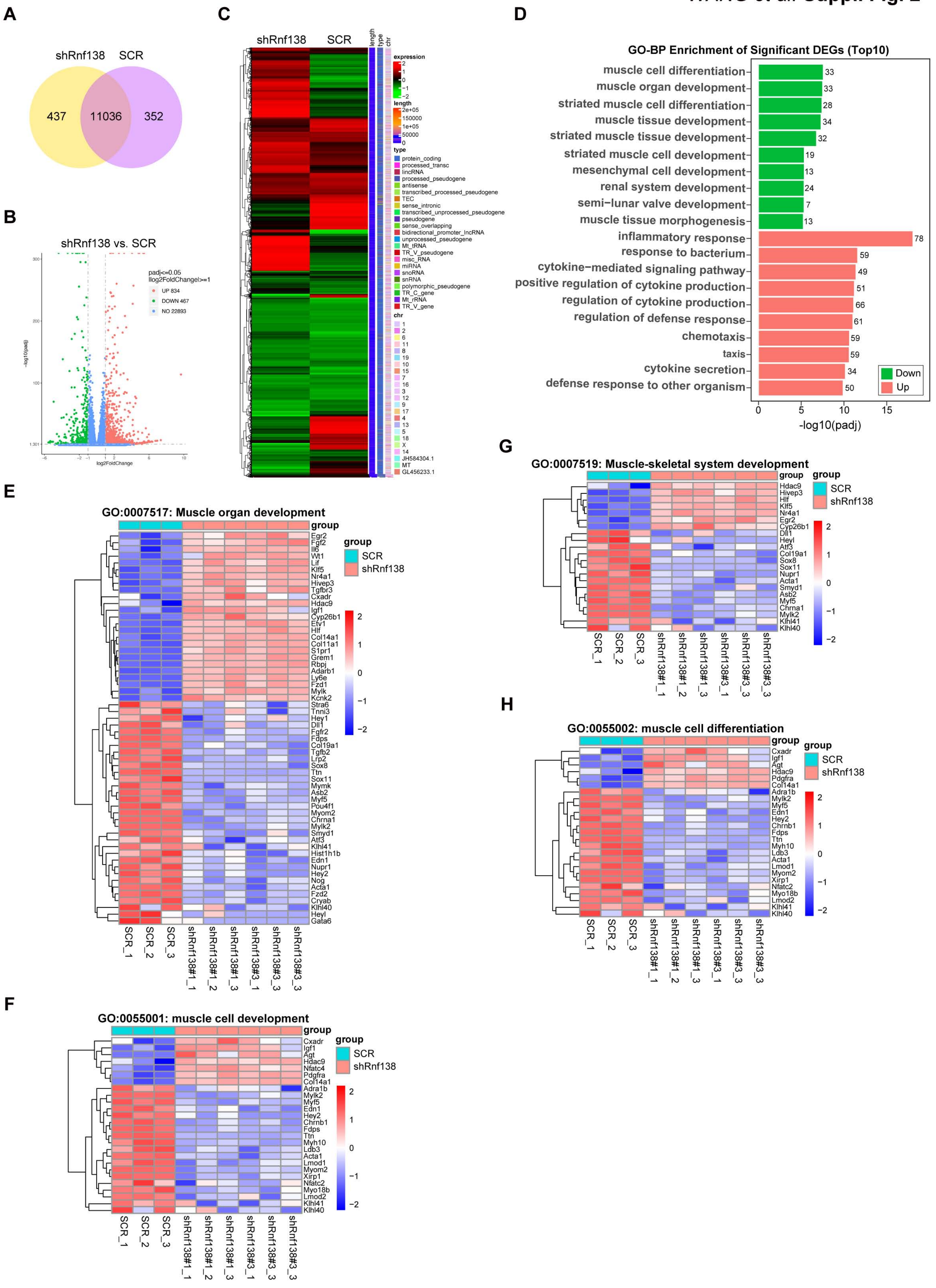

WANG et al. **Suppl. Fig. 3**

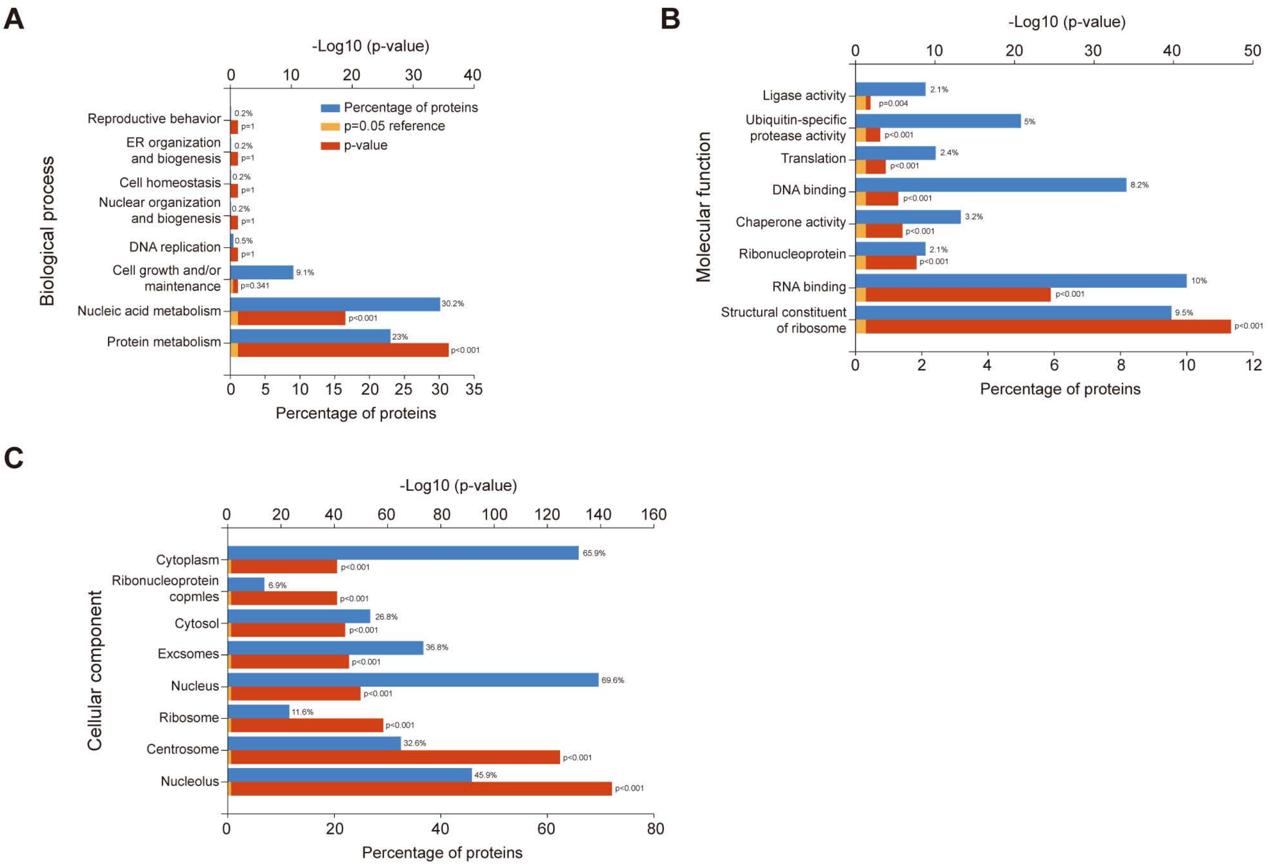

WANG et al. **Suppl. Fig. 4**

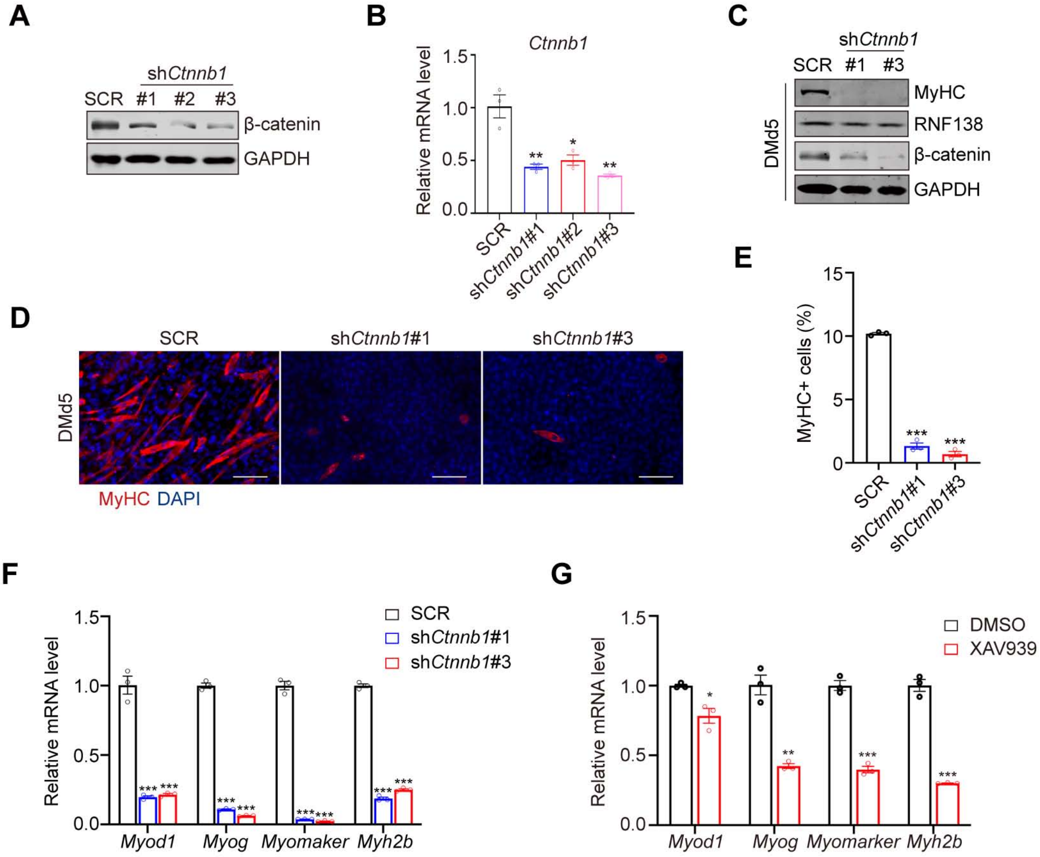

WANG *et al.* **Suppl. Fig. 5**

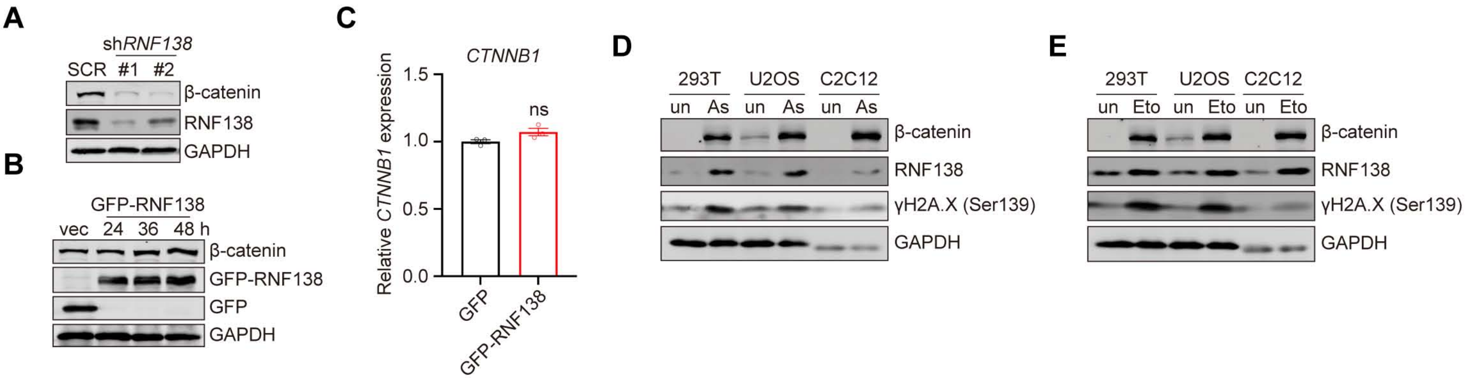

WANG et al. **Suppl. Fig. 6**

A

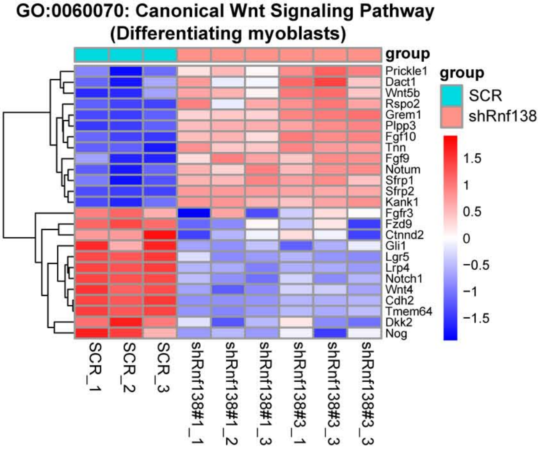

B

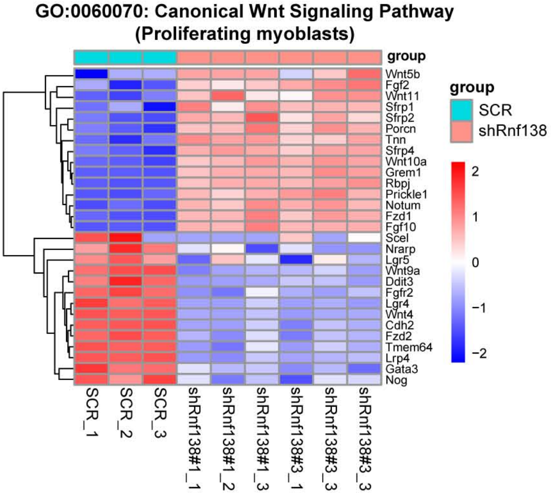

WANG et al. **Suppl. Fig. 7**

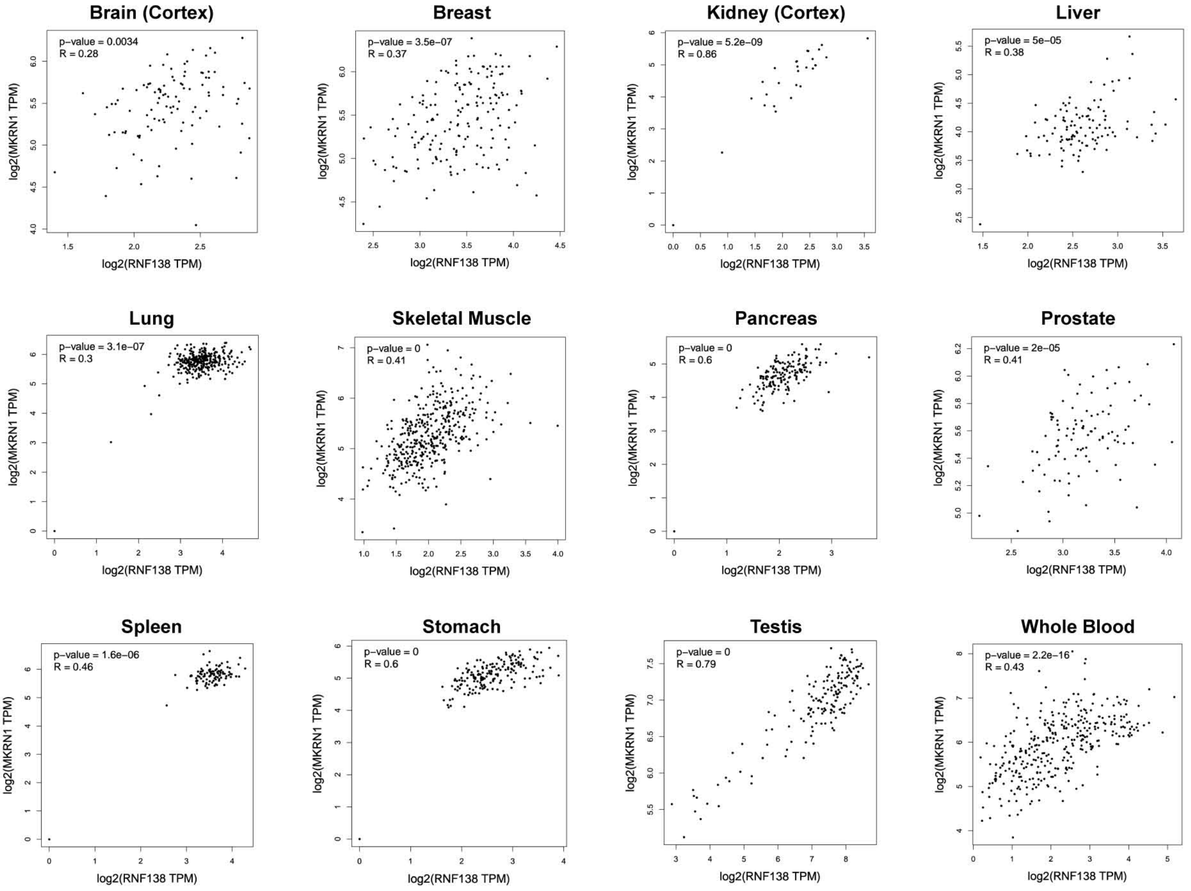

Supplement: Supplementary file 1 — Supplementary figures and tables. [file thnov15p4446s1.pdf]
